# Bile acid receptor Tgr5 prevents macrophage hyperinflammation during bacterial sepsis through metabolic and epigenetic silencing

**DOI:** 10.1016/j.isci.2025.113929

**Published:** 2025-11-05

**Authors:** Maria Reich, Tobias Franz, Haifeng C. Xu, Paulina Philippski, Jan Stindt, Sandra Freier, Anja Sammt, Kristina Schoonjans, Philipp A. Lang, Sascha Kahlfuß, Verena Keitel

**Affiliations:** 1 Department of Gastroenterology, Hepatology and Infectious Diseases, University Hospital Magdeburg, Medical Faculty, Otto-von-Guericke University, 39120 Magdeburg, Germany; 2 Institute of Molecular and Clinical Immunology, Medical Faculty, Otto-von-Guericke University, 39120 Magdeburg, Germany; 3 Institute of Medical Microbiology and Hospital Hygiene, Medical Faculty, Otto-von-Guericke University Magdeburg, 39120 Magdeburg, Germany; 4 Department of Molecular Medicine II, Medical Faculty, Heinrich-Heine University, 40225 Düsseldorf, Germany; 5 Institute of Biochemistry, Heinrich Heine University Düsseldorf, 40225 Düsseldorf, Germany; 6 Institute of Bioengineering, Faculty of Life Sciences, Ecole Polytechnique Fédérale de Lausanne, 1015 Lausanne, Switzerland; 7 Health Campus Immunology, Infectiology and Inflammation (GCI3), Medical Faculty, Otto-von-Guericke University Magdeburg, 39120 Magdeburg, Germany; 8 Center for Health and Medical Prevention, Otto-von-Guericke-University, 39120 Magdeburg, Germany

**Keywords:** natural sciences, biological sciences, immunology, microbiology

## Abstract

Tgr5 is a membrane-bound bile acid receptor that negatively regulates immune cells, although the molecular mechanisms behind this observation remain elusive. Here we report that Tgr5 is upregulated in macrophages during stimulation with *Listeria monocytogenes* and that Tgr5-deficient macrophages are hyperinflammatory and mice with myeloid Tgr5 deficiency are more susceptible to *Listeria monocytogenes* sepsis. Unexpectedly, Tgr5-deficient macrophages show reduced glycolysis and ATP citrate lyase expression, which cumulates in acetyl-CoA deficiency and impaired metabolic-epigenetic gene silencing, driving macrophages toward a hyperinflammatory phenotype during bacterial sepsis.

## Highlights

• Tgr5 expression in macrophages increases during bacterial infection
• Loss of myeloid Tgr5 drives hyperinflammatory responses and sepsis susceptibility
• Tgr5 maintains macrophage metabolism by supporting glycolysis and acetyl-CoA production
• Metabolic-epigenetic regulation by Tgr5 silences proinflammatory genes

## Graphical abstract

**Figure:**
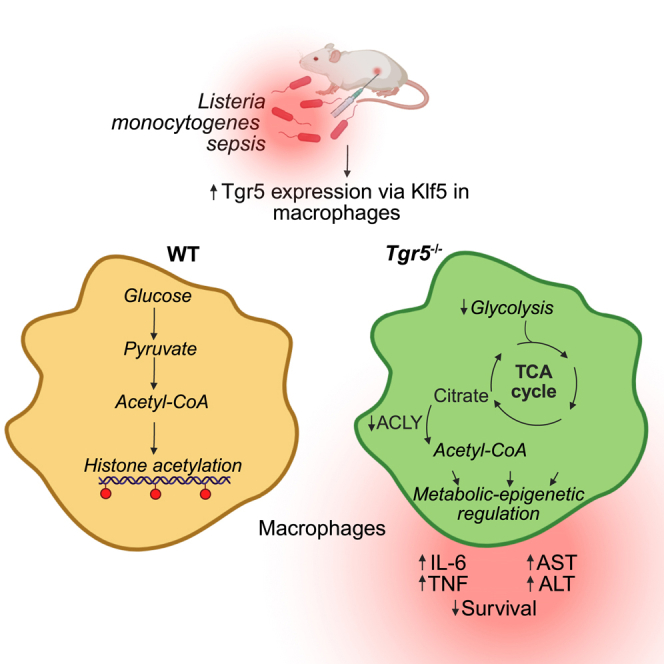

## Introduction

*Listeria monocytogenes* (*L.m.*) is a Gram-positive intracellular pathogen that causes foodborne infections in humans.^1^ While healthy adults usually suffer only from self-limiting gastroenteritis, immunocompromized and elderly patients can develop severe systemic infections involving the spleen, brain, and liver.^1^ Immunity to *L.m.* infection is mediated by T helper (Th) 1 cells and macrophages, whereas during *L.m.* sepsis, macrophage hyperactivation triggers a complex immunopathology driven by a severe cytokine storm.^2^

Tgr5 (Gpbar-1) is a membrane-bound G protein-coupled bile acid receptor expressed in several organs including spleen, bone marrow, intestine, and liver.^3^^,^^4^ Tgr5 expression and function have been implicated in the pathogenesis of metabolic, cardiovascular, liver, and pancreatic disorders as well as inflammatory bowel disease and gastrointestinal cancers. For instance, downregulation of TGR5 in biliary epithelial cells contributes to the pathogenesis of primary sclerosing cholangitis.^5^^,^^6^

Studies show that TGR5 upregulation during infection, particularly viral infections, is a protective mechanism that enhances antiviral immunity and reduces inflammation.^7^^,^^8^ Further, Tgr5-mediated bile acid signaling has been shown to have immunosuppressive effects on immune cells, including a reduction in the production of interleukin 6 (IL-6) and tumor necrosis factor (TNF) alpha by macrophages.^5^^,^^9^^,^^10^ In support of this, the production of proinflammatory cytokines after stimulation with lipopolysaccharide (LPS) is higher in macrophages isolated from Tgr5-deficient (*Tgr5*^*−/−*^) compared to wildtype (WT) mice.^11^ It is known that TGR5 inhibits NF-κB activation via cAMP signaling; however, the detailed mechanisms by which TGR5 is associated with the negative regulation of proinflammatory macrophages are yet incompletely understood.^11^^,^^12^

We show here that a myeloid-specific deletion of Tgr5 renders mice more susceptible to *L.m.* sepsis due to a hyperinflammatory phenotype and increased IL-6 and TNF production by Tgr5-deficient macrophages. Surprisingly, we observe that hyperinflammatory Tgr5-deficient macrophages do not show increased but reduced glycolysis. Impaired glycolysis in Tgr5-deficient macrophages cumulates in reduced ATP citrate lyase (Acly) expression and histone acetylation, which is important for silencing proinflammatory genes and controlling hyperinflammation during *L.m.* sepsis.

## Results

### Tgr5 expression is significantly upregulated in the liver and macrophages following infection with *L.m.* and treatment with LPS, and its transcription is regulated by Krüppel-like factor 5 (Klf5)

**Figure 1:**
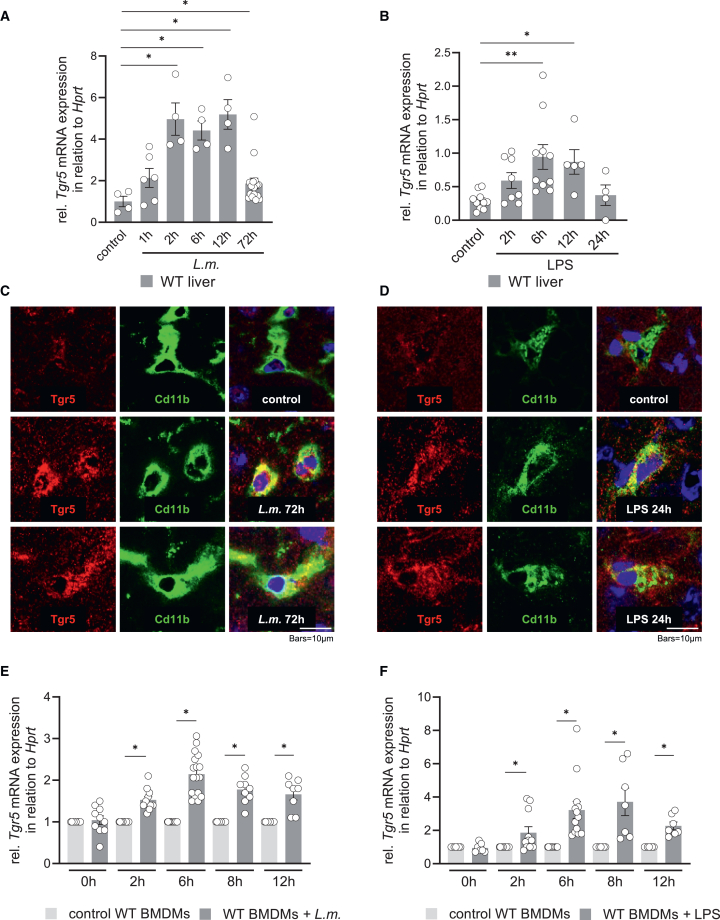
Infection with *L. monocytogenes* or injection of LPS leads to increased Tgr5 transcript and protein levels in wildtype mouse livers The relative *Tgr5* mRNA expression of livers from control and *L.m.*-infected (A) or LPS-injected (B) WT mice are shown in relation to *Hprt* mRNA expression (housekeeping gene). Data are expressed as mean ± SEM, statistical significance was determined by Mann-Whitney U test, ∗*p* < 0.05, ∗∗*p* < 0.01; compared to *Tgr5*^−/−^ mice under control or *L.m.-*infected or LPS-injected conditions; *n* = 4–16 for *L.m.-*infected and *n* = 4–10 for LPS-injected WT mice. Immunofluorescence staining of Tgr5 (red) in livers from control and *L.m.*-infected (C) or LPS-injected (D) WT mice. Immune cells in the liver were stained with an antibody against Cd11b (green) and nuclei were made visible using Hoechst (blue), LSM880×, 63× magnification, scale bars represent 10 μm. The relative *Tgr5* mRNA levels in isolated BMDMs treated with 1 × 10^6^ CFU/mL *L.m* (E) or incubated with LPS (100 ng/mL) (F) are shown relative to *Hprt* mRNA levels. Data are expressed as mean ± SEM. ∗ indicates a significant difference from the control condition (∗*p* < 0.05; Mann-Whitney U test, *n* = 5–16 for panel E; *n* = 4–12 for panel [F]). See also Figure S1.

To investigate whether Tgr5 expression is modulated by infection or inflammation, WT mice were injected with either *L.m.* or LPS. Quantitative RT-PCR analysis demonstrated a significant increase in *Tgr5* mRNA levels in the livers of WT mice following both *L.m.* infection (Figure 1A) and LPS treatment (Figure 1B). Immunofluorescence staining of liver sections confirmed an increase in Tgr5 protein levels, which were predominantly localized in immune cells (Cd11b^+^), following either *L.m.* infection (Figure 1C) or LPS exposure (Figure 1D). Similarly, Tgr5 mRNA expression was significantly elevated in bone marrow-derived macrophages (BMDMs) following stimulation with *L.m.* (Figure 1E) or LPS (Figure 1F), indicating that Tgr5 is upregulated in both hepatic tissue and immune cells during infection and inflammatory responses.

To elucidate the transcriptional regulation of Tgr5, we analyzed its putative promoter region and identified several potential binding sites for the transcription factor Klf5 (Figure S1A). Chromatin immunoprecipitation (ChIP) assays confirmed direct binding of Klf5 to the Tgr5 promoter region, as we observed enrichment of the promoter region following immunoprecipitation with Klf5-specific antibodies, in contrast to control IgG (Figures S1B and S1C). The functional role of Klf5 in regulating Tgr5 transcription was further evaluated using a luciferase reporter assays for the Tgr5 promotor region. Overexpression of Klf5 resulted in a dose-dependent increase in Tgr5 promoter activity (0.3 μg, 1.5 μg, 3.0 μg, and 4.5 μg), while mutations in the predicted Klf5-binding sites completely abolished this effect (Figures S1D and S1E).

To determine whether Klf5 is necessary for Tgr5 induction during infection, we performed siRNA-mediated knockdown of Klf5 in BMDMs. Silencing of Klf5 significantly impaired *L.m.-*induced *Tgr5* mRNA expression, demonstrating that Klf5 is essential for Tgr5 upregulation in response to *L.m.* (Figures S1F and S1G). Consistently, *Klf5* mRNA levels were transiently elevated in the livers of WT mice following *L.m.* infection (Figure S1H), suggesting that Klf5 expression plays a role in the early transcriptional response to infection.

Together, these data identify Klf5 as a direct and functional transcriptional regulator of Tgr5 and suggest that Tgr5 expression is dynamically regulated during bacterial infection and inflammation.

### Tgr5 in macrophages controls bacterial sepsis

**Figure 2:**
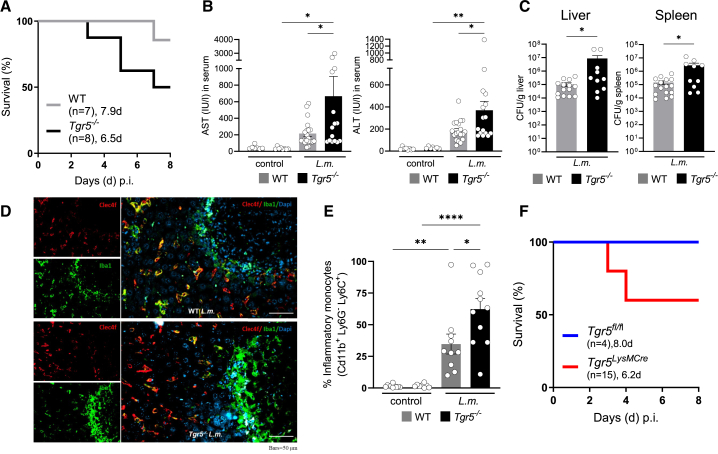
*Tgr5*^−/−^ mice were more susceptible to *Listeria monocytogenes* (*L.m.)* infection Wildtype (WT) and Tgr5 knockout (*Tgr5*^−/−^) mice were intravenously injected with *L. m.* (approx. 10^4^ CFU) and monitored daily until they reached the experimental endpoint criteria (A) Kaplan-Meier plot for experimental endpoint criteria following *L.m.* infection, analyzed by Log rank (Mantel-Cox) and Gehan-Breslow-Wilcoxon tests. (B) Serum levels of AST, and ALT. Data are expressed as mean ± SEM; *p* values were determined by one-way ANOVA (multiple comparisons), ∗*p* < 0.05, ∗∗*p* < 0.01; compared to *Tgr5*^−/−^ mice under control or *L.m.-*infected conditions. Sample sizes: *n* = 7–8 (WT), *n* = 7–8 (*Tgr5*^−/−^) for control; *n* = 19–20 (WT), *n* = 15–16 (*Tgr5*^−/−^*)* for *L. m*.-infected conditions. (C) Listeria titers were determined in the liver and spleen on day 3.5 in WT and *Tgr5*^−/−^ mice infected *i.v.* with 5 × 10^3^ CFU. Data are expressed as mean ± SEM; *p* values were determined by Mann-Whitney U test, ∗*p* < 0.05 compared to *Tgr5*^−/−^ mice under *L.m.-*infected conditions (*n* = 14–16 for WT, *n* = 10 for *Tgr5*^−/−^). (D) The livers of WT and *Tgr5*^−/−^ mice were stained for Kupffer cells (Iba1^+^Clec4f^+^) and proinflammatory monocyte-derived macrophages (Iba1^+^Clec4f^−^) (Zeiss Observer, 40× magnification, scale bars represent 50 μm). (E) Lymphocytes from the livers of control and *L.m.-*infected animals were enzymatically dispersed, stained for the common myeloid population (Cd11b^+^) as well as inflammatory monocytes (Cd11b^+^Ly6g^−^ Lys6c^+^), and analyzed by FACS. Data are expressed as mean ± SEM; *p* values were determined by one-way ANOVA (multiple comparisons), ∗*p* < 0.05, ∗∗*p* < 0.01; ∗∗∗∗*p* < 0.0001; compared to *Tgr5*^−/−^ mice under control or *L.m.-*infected conditions. Sample sizes: *n* = 10 (WT), *n* = 8 (*Tgr5*^−/−^) for control; *n* = 10 (WT), *n* = 11 (*Tgr5*^−/−^) for *L.m*.-infected conditions. (F) Kaplan-Meier plot for experimental endpoint criteria following *L.m.* infection of Tgr5-floxed control (*Tgr5*^*fl/fl*^) animals and myeloid cell-specific Tgr5 (*Tgr5*^*LysMCre*^) knockout mice (analyzed by log rank (Mantel-Cox) and Gehan-Breslow-Wilcoxon tests). See also Figure S2.

To investigate the role of Tgr5 during *L.m.* sepsis, Tgr5-deficient (*Tgr5*^−/−^) and WT littermate mice were intravenously injected with approximately 10^4^ CFU of *L.m.* After the infection, the mice were monitored daily. Following *L.m.* infection, half of the *Tgr5*^−/−^ mice reached the experimental endpoint at 3–7 days post infection, whereas 90% of WT mice survived the infection (Figure 2A). In line with a reduced overall survival, *Tgr5*^−/−^ mice showed significantly increased serum levels of aspartate aminotransferase (AST) and alanine aminotransferase (ALT) (Figure 2B) and a higher *L.m.* burden in the liver and spleen compared to WT mice (Figure 2C). In addition, we observed an increase in proinflammatory monocyte-derived macrophages (Iba1^+^Clec4f^+^) in the livers of *Tgr5*^−/−^ mice (Figure 2D). Confirming the latter observation, we detected significant higher frequencies of Cd11b^+^Ly6G^–^Lys6C^+^ monocytes in the livers of *Tgr5*^−/−^ compared to WT littermates (Figures 2E and S2A), while frequencies of neutrophils (Cd11b^+^Ly6G^+^Lys6C^−^) in the livers of *Tgr5*^−/−^ mice showed a trend toward upregulation but did not reach statistical significance (Figure S2B). Furthermore, *Tgr5*^−/−^ mice showed significant elevation in serum levels of the chemokine Ccl2 (Figures S2C and S2D), a ligand of the CCR2 receptor and surrogate parameter of systemic inflammation.^13^^,^^14^ To verify the role of Tgr5 in macrophages during sepsis, we generated myeloid cell-specific Tgr5 knockout (*Tgr5*^*LysMCre*^) mice and infected them with *L.m.* 40% of *Tgr5*^*LysMCre*^ animals reached the experimental endpoint criteria within eight days, whereas all of the *Tgr5*^*fl/fl*^ littermates survived the infection (Figure 2F). Together, these findings demonstrate that Tgr5 in monocyte-derived macrophages protects from hyperinflammation during bacterial sepsis.

To further investigate the role of Tgr5 in systemic inflammation beyond bacterial infection, we used an LPS-induced endotoxemia model as an additional method to mimic sepsis. WT and *Tgr5*^−/−^ mice were intraperitoneally injected with LPS (22.5 μg/g body weight), and the mice were monitored daily. In contrast to WT mice, which showed high survival rates, a significantly larger proportion of *Tgr5*^*−/−*^ mice reached the experimental endpoint criteria between 24 and 72 h (Figure S2E). Consistent with this increased susceptibility, *Tgr5*^*−/−*^ mice displayed elevated serum levels of the liver damage markers AST and ALT following LPS administration, compared to WT controls (Figure S2F). These findings mirror the phenotype observed in the *L.m.* infection model, indicating that Tgr5 is critical for maintaining tissue integrity and immune homeostasis during both bacterial and endotoxin-induced sepsis.

### Tgr5 prevents macrophages from hyperinflammation during bacterial sepsis

**Figure 3:**
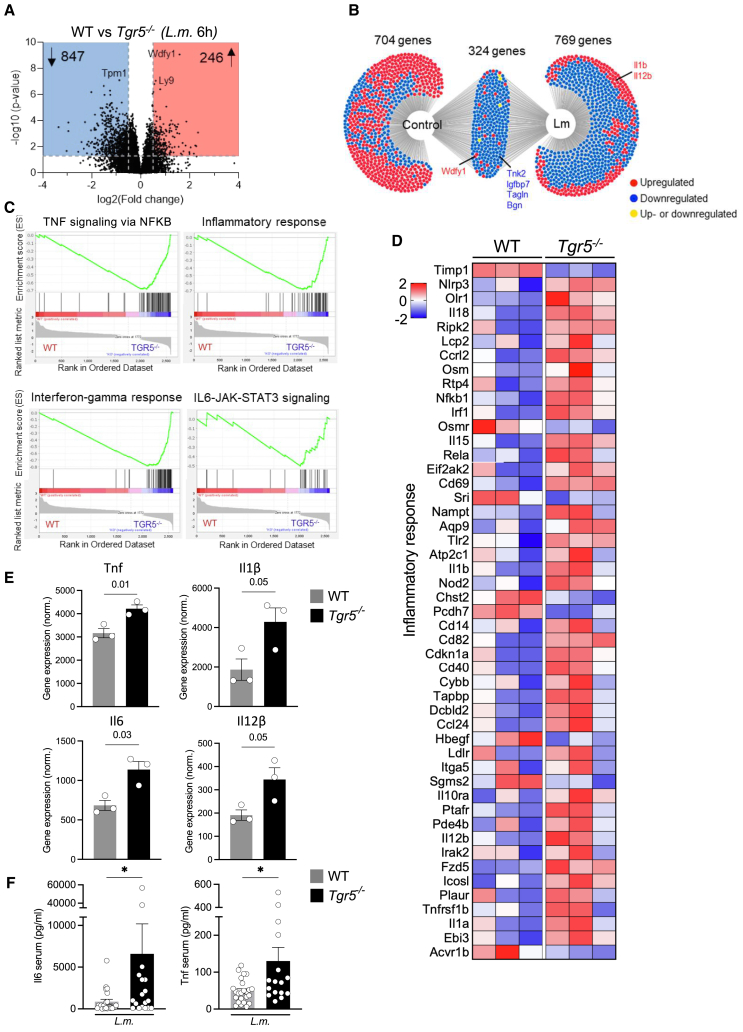
Tgr5 prevents inflammation in macrophages during bacterial sepsis by regulating inflammatory pathways (A) Volcano plot analysis of WT and Tgr5-deficient (*Tgr5*^−/−^*)* BMDMs treated with *L.m.* using bulk RNA sequencing. Highlighted in blue or red, respectively, are the differentially expressed genes (DEGs) with –log10(*p*-value)>1.3 and log2(fold change) > 0.5 or <0.5. The most significantly altered genes are marked; *n* = 3 biological replicates per genotype. (B) DiVenn diagram analysis of DEGs of untreated (control) and *L.m*.-treated Tgr5-deficient BMDMs. Selected genes are highlighted. (C) Enrichment plots for TNF signaling via NFKB, inflammatory response, interferon-gamma response, and IL6-JAK-STAT3 signaling. (D) Heatmap of DEGs associated with inflammation and inflammatory response in *L.m*.-infected BMDMs (6 h) from WT and *Tgr5*^−/−^ mice determined by RNA sequencing. The heatmap shows the relative minimum and maximum values for each gene (min/max). Numerical values are relative gene expression (*z*-scores). (E) Normalized gene expression from RNA seq data of inflammatory genes including Tnf, Il6, Il1b, and Il12b, ∗ = significant difference, (*p* < 0.05, based on RNA seq *p*-value). (F) Serum levels of Tnf and Il6 3.5 days post *L.m.-*infection in WT and *Tgr5*^−/−^ animals. Data are expressed as mean ± SEM; Mann-Whitney U test was used to determine statistical significance, defined as ∗*p* < 0.05 compared to *Tgr5*^−/−^ mice under *L.m.-*infected conditions (*n* = 16–24 for Tnf and *n* = 18–23 for Il6). See also Figure S3.

In order to investigate how Tgr5 influences transcriptional networks and the effector function of macrophages following bacterial sepsis, we differentiated BMDMs from WT and *Tgr5*^−/−^ mice, infected BMDMs with *L.m.* for 6 h, and performed bulk transcriptomic analyses. RNA sequencing revealed a total of 246 upregulated and 847 downregulated differentially expressed genes (DEGs) in Tgr5-deficient BMDMs infected with *L.m.* (Figures 3A and 3B). Gene set enrichment analysis indicated that the transcriptome of Tgr5-deficient BMDMs is positively correlated with proinflammatory signature pathways, including TNF signaling via NFKB (MM3860), inflammatory response (MM3890), interferon-gamma response (MM3878), and IL6-JAK-STAT3 signaling (MM3866) (Figure 3C). Ingenuity pathway analysis and BioCarta analysis showed an alteration of genes in Tgr5-deficient BMDMs that are associated with hepatic fibrosis signaling pathways and the cytokine network, respectively (Figure S3A). Among the top upregulated DEGs of Tgr5-deficient BMDMs were several proinflammatory cytokine genes (Figure 3D), including *Tnf*, *Il1b*, *Il6*, and *Il12b* (Figure 3E). The latter results were in line with higher Il6 and Tnf serum levels in *Tgr5*^−/−^ compared to WT littermate mice following *L.m.* sepsis (Figure 3F). In summary, the bile acid receptor Tgr5 prevents hyperinflammation of macrophages during bacterial sepsis.

### Tgr5-deficient BMDMs are hyperinflammatory but show reduced glycolytic capacity

**Figure 4:**
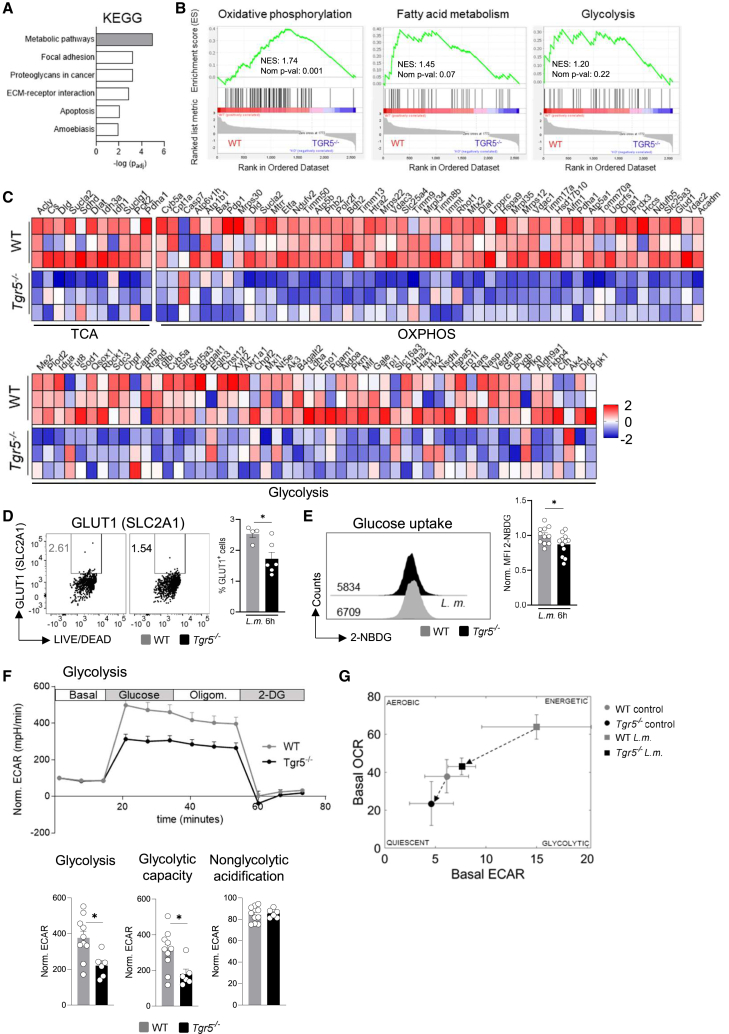
Tgr5-deficiency caused reduced glycolytic rate and glucose transporter type 1 (Glut1) expression in BMDMs after stimulation with *L.m* (A) KEGG analysis of upregulated pathways in Tgr5-deficient control and *L.m.-*stimulated BMDMs. (B) Enrichment plots for oxidative phosphorylation, fatty acid metabolism, and glycolysis. (C) Heatmap of TCA, oxidative phosphorylation and glycolysis-associated DEGs in BMDMs from WT and *Tgr5*^−/−^ mice infected with *L.m*. for 6h, as determined by RNA sequencing analysis. (D) Flow cytometric analysis of Glut1-positive BMDMs. Data are expressed as mean ± SEM; an unpaired Student’s *t* test was used to determine statistical significance, defined as ∗*p* < 0.05 compared to *Tgr5*^−/−^ mice under *L.m.-*infected conditions (*n* = 4–6). (E) Glucose uptake was determined using 2-NBDG and measured by FACS for the indicated groups. Unstimulated and stimulated murine BMDMs were incubated with *L.m.* (1 x 10^6^ CFU/mL) for 6 h. Data are expressed as mean ± SEM and were normalized to the mean of WT mice; an unpaired Student’s *t* test was used to determine statistical significance, defined as ∗*p* < 0.05 compared to *Tgr5*^−/−^ mice under *L.m.-*infected conditions (*n* = 11–12). (F) The glycolytic activity of *L.m.-*infected BMDMs from WT and *Tgr5*^−/−^ mice was determined by Seahorse analysis (ECAR: extracellular acidification rate). Bar graphs show glycolysis, glycolysis capacity, and non-glycolytic acidification from data in (F). Data are expressed as mean ± SEM; an unpaired Student’s *t* test was used to determine statistical significance, defined as ∗*p* < 0.05 compared to *Tgr5*^−/−^ mice under uninfected conditions (*n* = 6–10). (G) Energy map of basal oxygen consumption rate (OCR) plotted against basal ECAR. See also Figure S4.

*Tgr5*^−/−^ and *Tgr5*^*LysMCre*^ mice suffered from severe *L.m.* sepsis and Tgr5-deficient macrophages were prone to a hyperinflammatory phenotype (Figures 3 and 4). However, the mechanisms behind these observations still remained unknown. In this context, KEGG pathway analyses of all DEGs in Tgr5-deficient BMDMs following *L.m.* infection indicated that most of these genes belonged to metabolic pathways (Figure 4A), specifically oxidative phosphorylation (MM3893), fatty acid metabolism (MM3892), and glycolysis (MM3894) (Figure 4B). Here, especially genes encoding enzymes of the tricarboxylic acid (TCA) cycle, oxidative phosphorylation, and glycolysis appeared markedly downregulated in Tgr5-deficient BMDMs (Figure 4C). However, oxidative phosphorylation (Figures S4A and S4B), ATP production (Figure S4C), and the expression of most electron transfer chain (ETC) proteins (Figures S4F–S4H) as well as the uptake of short and long chain fatty acids (Figures S4D and S4E) were unaltered in Tgr5-deficient BMDMs compared to WT BMDMs. In contrast, Tgr5-deficient BMDMs showed significantly reduced expression of the glucose transporter Glut-1 (Slc2a1) (Figure 4D) and a diminished glucose uptake (Figure 4E), which cumulated in reduced glycolysis and glycolytic capacity (Figures 4F and 4G). Vice versa, stimulation with a Tgr5 agonist in WT BMDMs under *L.m.*-stimulated conditions led to a significant increase in glucose uptake compared with conditions without a Tgr5 agonist (Figures S5G and S5H).

### Pharmacological inhibition of Acly exacerbates macrophage cytokine production

**Figure 5:**
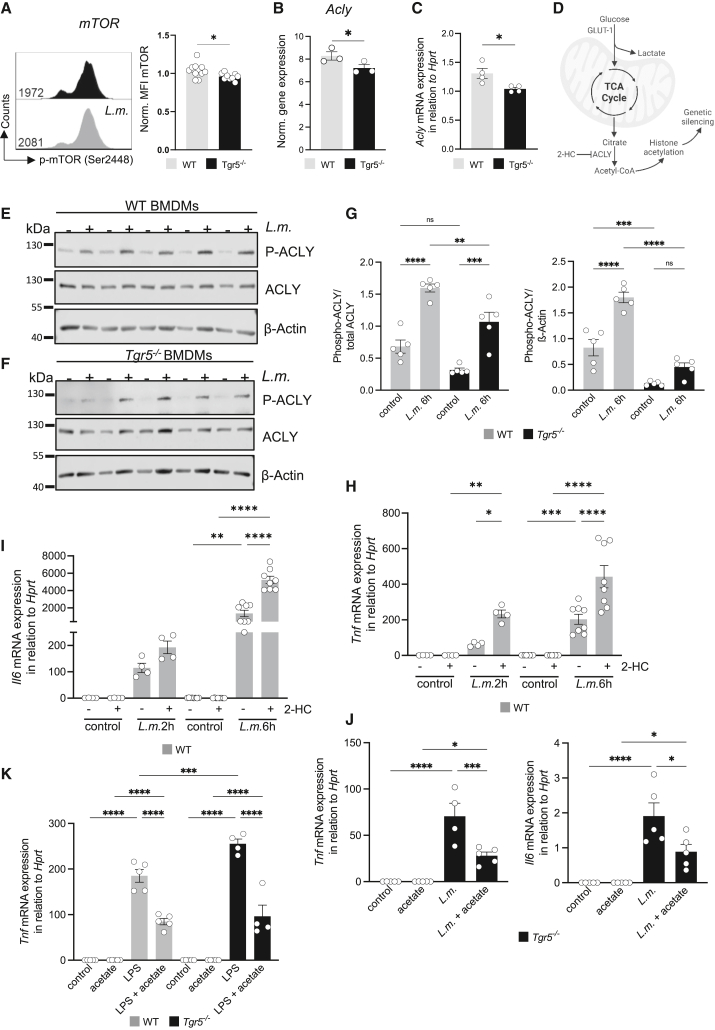
Pharmacological inhibition of ACLY exacerbates macrophage cytokine production (A) Expression of phospho-mTOR (Ser2448) in WT and *Tgr5*^−/−^ BMDMs under *L.m.-*stimulated conditions measured by flow cytometry. Data are shown as mean ± SEM and were normalized to the mean of WT cells; *p* values were determined by unpaired Student’s *t* test, ∗*p* < 0.05 (*n* = 15–17 for WT and *Tgr5*^*−/−*^). (B) Normalized gene expression from RNA sequencing data of ATP citrate lyase (Acly) expression, ∗*p* < 0.05, based on RNA sequencing *p*-value. (C) *Acly* mRNA expression was analyzed in relation to *Hprt* in *L.m.*-infected BMDMs after 2 h. Data are expressed as the mean ± SEM; *p* values were determined by Mann-Whitney U test, ∗*p* < 0.05 compared to *Tgr*5^−/−^ mice under *L.m.-*infected conditions (*n* = 4 for WT and *Tgr*5^−/−^). (D) Effect of 2-hydroxycitrate (2-HC) to inhibit ATP-citrate lyase (Acly) in WT BMDMs. Western blot analysis was performed to assess the levels of phosphorylated ATP-citrate lyase (*p*-ACLY) and total ACLY in primary bone marrow-derived macrophages (BMDMs) isolated from (E) wildtype (WT; *n* = 5) and (F) Tgr5 knockout (*Tgr*5^−/−^; *n* = 5) mice. (G) Densitometric quantification was performed to evaluate the relative expression of *p*-ACLY (Ser455), normalized to either total ACLY or β-Actin. Data are presented as mean ± SEM. *p* values were determined using one-way analysis of variance (ANOVA); ∗∗*p* < 0.01, ∗∗∗*p* < 0.001, ∗∗∗∗*p* < 0.0001. RT-PCR analysis of *Tnf* (H) and *Il6* (I) mRNA expression in relation to *Hprt* in the presence of Acly-inhibitor (2-HC) in *L.m.*-infected BMDMs for 2 and 6 h. BMDMs were pre-treated with 5mM 2-hydroxycitrate (2-HC) for 30min before stimulation with *L.m*. Data are expressed as mean ± SEM, *p* values were determined by one-way ANOVA (multiple comparisons), ∗*p* < 0.05, ∗∗*p* < 0.01, ∗∗∗*p* < 0.001, ∗∗∗∗*p* < 0.0001 compared to control (*n* = 4) or *L.m.-*infected conditions (*n* = 4–8) with or without the treatment with 5 mM 2-HC for 2 h or 6 h. (J) RT-PCR analysis of *Tnf* and *Il6* mRNA expression relative to *Hprt* in *Tgr*5^−/−^ BMDMs stimulated with *L.m.* for 2 h followed by treatment with 10 mM acetate for an additional 2 h. Data are presented as mean ± SEM. *p* values were determined by one-way ANOVA; ∗*p* < 0.05, ∗∗*p* < 0.01, ∗∗∗*p* < 0.001, ∗∗∗∗*p* < 0.0001. (K) RT-PCR analysis of *Tnf* expression relative to *Hprt* in BMDMs stimulated with LPS for 2 h followed by treatment with 10 mM acetate for an additional 2 h. Data are presented as mean ± SEM. *p* values were determined by one-way ANOVA; ∗∗∗*p* < 0.001, ∗∗∗∗*p* < 0.0001. See also Figure S5.

Tgr5-deficient BMDMs were hyperinflammatory but, in contrast, showed reduced glycolytic capacity. *Tgr5*^*−/−*^ BMDMs showed significantly decreased expression of phosphorylated mammalian target of rapamycin (*p*-mTOR, Ser2448), a key protein kinase regulating cellular metabolism and proliferation (Figures 5A and S5B), which is in line with previous studies.^15^ However, the expression of phosphorylated AMP-activated protein kinase, peroxisome proliferator-activated receptor gamma (PPARγ), phosphorylated protein kinase B, and cellular myelocytomatosis oncogene were unaltered in Tgr5-deficient BMDMs compared to WT BMDMs (Figures S5C–S5F). One of the top downregulated genes in *L.m.-*infected Tgr5-deficient BMDMs was Acly encoding the enzyme ATP-citrate lyase (Acly), which catalyzes the release of cytosolic acetyl-CoA from citrate (Figures 5B–5D). Acetyl-CoA serves as a molecular rheostat that links metabolic activity to cellular function.^12^ First, cytosolic acetyl-CoA serves as a substrate for the biosynthesis of fatty acids and sterols, promoting lipid synthesis. In addition, acetyl-CoA is essential for histone modification and thereby gene regulation.^13^ To investigate the role of Acly in the regulation of inflammatory gene expression in the context of Tgr5 deficiency, we next assessed the phosphorylation status of ACLY, a key indicator of its enzymatic activity. Western blot analysis demonstrated a significant decrease in phosphorylated ACLY (p-ACLY, Ser455), an indicator of its activation, in *Tgr5*^−/−^ BMDMs compared to WT following *L.m.* infection (Figures 5E–5G). This finding suggests that Tgr5 is essential for the efficient activation of ACLY under inflammatory conditions. To prove whether reduced acetylation affects gene expression of proinflammatory cytokines, we cultured WT BMDMs in the absence and presence of 2-hydroxycitrate (2-HC), a competitive inhibitor of Acly, followed by gene expression analyses of Tnf and Il6 (Figures 5H and 5I). Importantly, 2-HC significantly increased *Tnf* and *Il6* gene expression in WT BMDMs. ACLY inhibition promotes tumor immunity and suppresses liver cancer,^16^ demonstrating that ACLY blockade enhances immunogenicity, a finding that complements our observation that Acly activity links metabolic state to inflammatory gene control and may influence sepsis outcomes. We next hypothesized that supplementation of exogenous acetate, a metabolic precursor of acetyl-CoA, could restore intracellular acetyl-CoA levels and attenuate the hyperinflammatory response. Indeed, treatment of *L.m.-*stimulated BMDMs with acetate led to a significant reduction in the expression of *Tnf* and *Il6* mRNA in *Tgr5*^−/−^ BMDMs (Figure 5J). Subsequently, we investigated the anti-inflammatory effects of acetate on cytokine expression following LPS stimulation. RT-PCR analysis demonstrated that acetate treatment significantly suppressed LPS-induced *Tnf* and *Il6* expression in both WT and *Tgr5*^−/−^ BMDMs (Figures 5K and S5A).

Together, our results indicated that Tgr5 is important for the glycolytic capacity and genetic silencing of proinflammatory genes to prevent hyperinflammation during bacterial sepsis.

## Discussion

Tgr5 signaling in macrophages has been shown to negatively regulate inflammatory cytokine production.^5^^,^^9^^,^^10^ In this context, *in vitro* studies have demonstrated that macrophages from *Tgr5*^−/−^ mice produce higher amounts of proinflammatory cytokines upon LPS stimulation.^11^ In our current study, we confirm and extend on these studies as we find that *Tgr5*^*LysMCre*^ mice are prone to *L.m.* sepsis. We further find that conditional deletion of *Tgr5* induces a proinflammatory gene signature and increased cytokine production by macrophages. However, in contrast to the observation that Tgr5-deficient macrophages are functionally hyperinflammatory, we, unexpectedly, detect reduced glycolysis and glycolytic capacity. In line with this, studies demonstrated that macrophages in the lungs of patients with severe COVID-19 exhibit reduced activity in most metabolic processes, such as TCA cycle, compared to those from healthy individuals, whereas macrophages with mild COVID-19 show an increase in the majority of essential metabolic pathways.^17^ This suggests that severe infections may cause a hyperinflammatory state coupled with metabolic impairment. Zhao et al. (2022) show partial rescue with PPARγ stimulation or amino acid supplementation for this metabolic impairment, but the efficacy of such treatment in *L.m.* sepsis remains to be determined.

Mechanistically, our data suggest that reduced glycolysis and the lack of Acly-derived acetyl-CoA in Tgr5-deficient macrophages mediates epigenetic silencing of proinflammatory genes via reduced histone acetylation.

In support of this notion, supplementation of exogenous acetate attenuated the hyperinflammatory response and inhibition of Acly-derived acetyl-CoA generation reverses the Tgr5-dependent proinflammatory cytokine suppression. Our results are in line with recent studies suggesting that a genetic deletion of ACLY renders macrophages toward higher IL-1b, TNF, and CCL2 production^18^ and demonstrate that the bile acid receptor Tgr5 in macrophages is essential to prevent hyperinflammation during bacterial sepsis through metabolic-epigenetic gene regulation. Recently, it was shown that genetic ACLY deficiency or ACLY inhibition leads to increased immunogenicity and, consequently, inhibits the development/progression of hepatocellular carcinoma,^16^ supporting the notion that Tgr5 modulates cellfunction via metabolic-epigenetic pathways. Tgr5 signaling largely suppresses inflammatory transcription via cAMP/PKA axis, impacting NF-κB,^11^^,^^12^^,^^19^ MAPK,^19^^,^^20^^,^^21^ and metabolic-epigenetic regulators such as ACLY/acetyl-CoA, which in turn might influence C/EBPβ and mTOR-driven transcription in macrophages.

Preclinical studies have demonstrated promising efficacy of selective Tgr5 agonists in the treatment of metabolic and inflammatory disorders; however, side effects, such as gallbladder filling, still restrict their clinical application, highlighting the need for further optimization.^22^

### Limitations of the study

While this study shows that Tgr5 signaling modulates ACLY expression, the precise mechanism, such as cAMP-PKA-axis or alternative pathways, remains to be determined and whether regulation is transcriptional or post-translational is unclear. Seahorse extracellular flux analyses show reduced glycolysis (extracellular acidification rate) with largely unchanged OXPHOS (oxygen consumption rate) in Tgr5-deficient BMDMs; however, the reasons why OXPHOS remains stable despite ACLY’s role in anaplerosis require further investigation. Additionally, performing *in vivo* rescue experiments in *Tgr5*^*−/−*^ mice to test reversibility of hyperinflammation with acetyl-CoA precursors or ACLY activators would strengthen our findings. Finally, LPS-stimulated findings may not fully reflect *Listeria*-specific TLR/NOD signaling; validation with heat-killed *Listeria* or purified Listeriolysin O toxin would strengthen generalizability.

## Resource availability

### Lead contact

Requests for further information and resources should be directed to and will be fulfilled by the lead contact, Verena Keitel (verena.keitel-anselmino@med.ovgu.de).

### Materials availability

This study did not generate new unique reagents.

### Data and code availability

• RNA sequencing data are available on Gen Expression Omnibus (GEO) under GSE309415. Data of Differentially Expressed Genes (DEGs) and Gene Set Enrichment Analysis (GSEA) using RNA sequencing data are available on https://doi.org/10.5281/zenodo.17186280 and https://doi.org/10.5281/zenodo.17186503, respectively. The remaining raw data supporting the findings of this study can be made available by the lead contact upon reasonable request.

## Acknowledgments

We thank PD Dr. rer. nat Ludger Klein-Hitpaß from the Institute of Cell Biology (Cancer Research), Faculty of Medicine, University of Duisburg-Essen, Essen, Germany, for performing the RNA sequencing. We also acknowledge Dr. Rene Scholtysik from the same institute for his assistance with the processing of RNA sequencing data. We thank Dr. Chakravarthi Chintalapati and Christina Wöhler for support with the TGR5/Tgr5 promotor analysis. This work was supported by the 10.13039/501100001659Deutsche Forschungsgemeinschaft (DFG, German Research Foundation), grant no. 361210922/GRK2408, (project 12) and the 10.13039/501100003042Else Kroener-Fresenius-Stiftung (2023_EKEA.128) to S.K. and by the 10.13039/501100001659Deutsche Forschungsgemeinschaft (DFG, German Research Foundation), grant no. 361210922/GRK2408, (project 11) to V.K.

## Author contributions

V.K. and S.K., together with M.R. and T.F., designed the study and planned the experiments. M.R. and T.F. drafted and S.K., V.K., M.R., and T.F. wrote the manuscript. M.R., T.F., H.C.X., P.P., J.S., S.F., A.S., and K.S. conducted experiments. J.S. and P.A.L. helped discussing and interpretation of the data. All authors approved the final version of the manuscript.

## Declaration of interests

The authors have declared that no conflict of interest exists.

## STAR★Methods

### Key resources table

**Table:** 

REAGENT or RESOURCE	SOURCE	IDENTIFIER
**Antibodies**		

Alexa Fluor 647-AffiniPure Donkey Anti-Goat IgG (H+L)	Jackson ImmunoResearch Labs	Cat# 705-605-147, RRID:AB_2340437
ATP-Citrate Lyase	Cell Signaling	Cat# 13390
ß-Actin	Sigma	Cat# A5441
BTEB2 (G-7)	Santa Cruz Biotechnology	Cat# sc-398470
CD11b	eBioscience	Cat# 14-0112-82; RRID:AB_467108
CD11b Monoclonal Antibody (M1/70), APC-eFluor™ 780, eBioscience	Thermo Fisher Scientific	Cat# 47-0112-82, RRID:AB_1603193
CLEC4F/CLECSF13	R&D Systems /Biotechne	Cat# AF2784; RRID:AB_2081339
c-Myc	Cell signaling Technology	13987
Cy3-AffiniPure Donkey Anti-Rabbit IgG (H+L)	Jackson ImmunoResearch Labs	Cat# 711-166-152, RRID:AB_2313568
Cy3-AffiniPure F(ab')2 Fragment Donkey Anti-Mouse IgG (H+L)	Jackson ImmunoResearch Labs	Cat# 715-166-150, RRID:AB_2340816
F4/80	Thermo Fisher Scientific	Cat# 14-4801-82; RRID:AB_467558
Fc-block anti CD16/32	BioLegend	Cat# 101302
Fluorescein (FITC)-AffiniPure F(ab')2 Fragment Donkey Anti-Rat IgG (H+L)	Jackson ImmunoResearch Labs	Cat# 712-096-153, RRID:AB_2340654
Glut-1	Thermo Fisher Scientific	Cat# MA5-31960, RRID:AB_2809254
IBA1	FUJIFILM Wako Pure Chemical Corporation	Cat# 019-19741, RRID:AB_839504
Goat anti-rabbit AF750	Invitrogen	Cat# A21039
Goat anti-Rabbit IgG (H+L) Highly Cross-Adsorbed Secondary Antibody, Alexa Fluor™ Plus 488	Thermo Fisher Scientific	Cat# A32731, RRID:AB_2633280
mouse anti-Klf5, BTEB2 (G-7)	Santa Cruz Biotechnology	Cat# sc-398470-x
Ly-6C Monoclonal Antibody (HK1.4), PerCP-Cyanine5.5, eBioscience	Thermo Fisher Scientific	Cat# 45-5932-82, RRID:AB_2723343
Ly-6G/Ly-6C Monoclonal Antibody (RB6-8C5), FITC, eBioscience	Thermo Fisher Scientific	Cat# 11-5931-82, RRID:AB_465314
2’NBDG FITC	Invitrogen	Cat# N13195
normal mouse IgG	Santa Cruz Biotechnology	Cat# sc-2025
Phospho-AMPK (Thr172)-FITC Conjugated	Bioss Antibodies	Cat# bs-4002R
PPAR Gamma Polyclonal Antibody, PE-Cy7 Conjugated	Bioss Antibodies	Cat# bs-4590R
Phospho-Akt (Ser473)-PE-Cy7 Conjugated	Cell signaling Technology	Cat# 88106
Phospho-ATP-Citrate Lyase	Cell Signaling	Cat# 4331
Phospho-mTOR (Ser2448)-PE Conjugated	Invitrogen	Cat# 12-9718-42
TGR5 Polyclonal Antibody	Thermo Fisher Scientific	Cat# PA5-23182
Total OXPHOS Rodent WB AB Cocktail	Abcam	Cat# Ab110413

**Bacterial and virus strains**		

*Listeria monocytogenes* (Murray et al.) Pirie	ATCC	Cat# 43251

**Chemicals, peptides, and recombinant proteins**		

BD BBL™ Brain Heart Infusion Agar	BD	Cat# 211065
BD BBL™ Brain Heart Infusion Broth	BD	Cat# 211059
DMEM, low glucose, pyruvate	Thermo Fisher Scientific	Cat# 31885049
DPBS	PAN-Biotech	Cat# P04-36500
EDTA	ROTH	Cat# CN06.2
FCS	Sigma	Cat# S0615
Forskolin	Sigma-Aldrich	Cat# F3917
Hydroxycitric acid tripotassium salt monohydrate	Santa Cruz Biotechnology	Cat# sc-490555; CAS Number: 232281-44-6
Lipopolysaccharide aus *Salmonella typhimurium*	Sigma-Aldrich	Cat# L7261
Mouse M-CSF Recombinant Protein, PeproTech®	Thermo Fisher Scientific	Cat# 315-02
OptiMEM	Thermo Fisher Scientific	Cat# 51985-034
Penicillin-Streptomycin (10.000 U/ml)	Thermo Fisher Scientific	Cat# 15140122
Pierce™ 16% Formaldehyde, Methanol-free	Thermo Fisher Scientific	Cat# 28908
Phusion High Fidelity DNA polymerase	Thermo Fisher Scientific	Cat# F530L
Polyethylenimine	Sigma-Aldrich	Cat# 408727
Protein Assay Day Reagent Concentrate	Bio-Rad	Cat# 5000006
RO5527239 ((R,E)-1-(4-(3-(hydroxyimino)-3-(2-methylpyridin-4-yl)-1-o-tolylpropyl)phenyl)piperidine-4-carboxylic acid)	US-Pat.-Appl. US-20120010190	provided by F.Hoffmann-La Roche Ltd. (Basel, Switzerland)
ROTI®Histofix 4% Formaldehyde	Roth	Cat# P087.1
RPMI^-Glc^	PAN-Biotech	P04-16530
siGLO Red Transfection Indicator	Dharmacon Reagents	Cat# D-001630-02-05
Sodium-Acetate	Roth	Cat# 6773.2
Taurolithocholic acid (TLC)	Sigma-Aldrich	Cat# 6042-32-6
Trypsin 0.05 %/EDTA 0.02 % in DPBS, w/o: Ca and Mg	PAN Biotechne	Cat# P10-023100

**Critical commercial assays**		

ATP Colorimetric/Fluorometric Assay Kit	Merck	Cat# Mak190
Dual Luciferase Assay Reporter Assay-System	Promega	Cat# E1910
eBioscience™ FOXP3/ Transcription Factor Staining Buffer Set	Invitrogen	00-5523-00
Millipore ChIP Assay Kit	Merck Millipore	Cat# 17-295
MILLIPLEX® Mouse Cytokine/Chemokine Magnetic Bead Panel - Immunology Multiplex Assay	Merck Millipore	Cat# MCYTOMAG-70K
QIAquick PCR Purification Kit	Qiagen	Cat# 28106
SF Cell Line 4D-Nucleofector® X Kit S	Lonza	Cat# V4XC-2032

**Deposited data**		

Raw and analyzed RNA sequencing data	This paper	GEO: GSE309415
Data of Differentially Expressed Genes (DEGs)	This paper	https://doi.org/10.5281/zenodo.17186280
Gene Set Enrichment Analysis (GSEA)	This paper	https://doi.org/10.5281/zenodo.17186503,

**Experimental models: Cell lines**		

RAW 264.7	in-house stock	N/A
Human embryonic kidney 293 (HEK-293)	in-house stock	N/A

**Experimental models: Organisms/strains**		

Mouse WT and *Tgr5*^*-/-*^*:* BL/6.129-TgH-(Gpbar1)	Merck Sharp & Dohme Corp. New Jersey, USA	N/A
Mouse *Tgr5*^*LysMCre*^: C57BL/6J;129-TgH(Tgr5^fl/f^l) Lyz2^tm1(cre)Ifo^	Generated in-house	N/A
Mouse *Tgr5*^*fl/fl*^ : C57BL/6J;129Sv-TgH(Tgr5^fl/fl^)	Kristina Schoonjans (Ecole Polytechnique Fédérale in Lausanne (EPFL), Switzerland)	N/A

**Oligonucleotides**		

siGENOME SMARTpool murine Klf5 siRNA	Horizon	D-062477-01
siGENOME SMARTpool murine Klf5 siRNA	Horizon	D-062477-02
siGENOME SMARTpool murine Klf5 siRNA	Horizon	D-062477-03
siGENOME SMARTpool murine Klf5 siRNA	Horizon	D-062477-04
siGENOME Non-targeting siRNA #1	Horizon	D-001210-01-05
siGENOME Non-targeting siRNA #3	Horizon	D-001210-03-05
siGENOME Non-targeting siRNA #3	Horizon	D-001210-05-05
Tgr5promfor: ACGGTACCAGCAGTCAGGATGTGCCAC	This paper	N/A
Tgr5promrev: GTGCTAGCGAGTCCAAGCTGAAGGACC.	This paper	N/A
Tgr5 promotor Chip eluates 334bp-for (5'-CAAGAGGGTTCTCAGAGG TAAG-3')334bp-rev (5' TCTGCGGA TCAAAGAGTCAC-3')	This paper	N/A
Chip eluates 524bp-for (5'-CCCACCTCCATCTTACATC-3') and 524bp-rev (5'-TCACATCAGCCACTCTTG-3'),	This paper	N/A
TaqMan gene expression assays, see Table S2	Thermo Fisher Scientific, Applied Biosystems	Cat # 4331182

**Software and algorithms**		

Axiovision Software	Becton Dickinson, Ashland, OR	4.8.3 SP3
Agilent Seahorse Analytics	Agilent Technologies	Agilent Seahorse Wave, Version 2.6.1
DAVID Bioinformatics	U. S. National Institutes of Health	Database for Annotation, Visualization, and Integrated Discovery (DAVID); v2024q4
FlowJo	Becton Dickinson, Ashland, OR	10.0.7
GraphPad Prism	GraphPad Software Inc., San Diego, CA	10.4.0
QIAGEN Ingenuity Pathway Analysis (QIAGEN IPA)	QIAGEN Bioinformatics	9.5.3
Trimmomatic software	Bolger et al.^23^; https://doi.org/10.1093/bioinformatics/btu170	0.30
ZEISS ZEN software	Carl Zeiss AG, Oberkochen, DE	10.0.19045 Build 19045

**Other**		

4D-Nucleofector® X Unit	Lonza	Cat# AAF-1003X
FACSAria III cytometer	BD Biosciences	Heidelberg, Germany
LSM 880 confocal microscope	Zeiss	
Zeiss Axio Observer Z1/7	Zeiss	

### Experimental model and study participant details

#### Animals and animal procedures

*Tgr5*^*-/-*^ mice were described previously.^5^ Tgr5 floxed mice (*Tgr5*^*fl/fl*^) were kindly provided by Professor Kristina Schoonjans (Ecole Polytechnique Fédérale in Lausanne (EPFL), Switzerland). *Tgr5*^*LysMCre*^ mice were bred in-house at the Central Institution for Animal Research and Scientific Animal Welfare (ZETT), Düsseldorf. *Tgr5*^*-/-*^, *Tgr5*^*fl/fl*^ and *Tgr5*^*LysMCre*^ mice were all maintained on a C57BL/6 background. Breeding was carried out with heterozygous animals to obtain littermate controls. Mice had access to water and food *ad libitum* and were maintained on 12 h light/dark cycles. Animal experiments were approved by North Rhine-Westphalia's Ministry for Climate Protection, Environment, Agriculture, Nature Conservation and Consumer Protection (LANUV) Animal Welfare Committee, Germany (AK: 84.-02.04.2011.A275, 84-02.04.2015.A314 and 81-02.04.2018.A131).

#### *Listeria monocytogenes* infection

*Listeria monocytogenes (L.m.)* (Strain: 43251; ATCC, Wesel, Germany) were maintained in brain heart infusion (BHI; BD Diagnostics, USA) as previously described.^24^^,^^25^ For infection studies, age-matched (12-16 weeks) male *Tgr5*^*-/-*^, *Tgr5*^*fl/fl*^ and *Tgr5*^*LysMCre*^ mice were injected intravenously (*i.v.)* via the tail vein with a target dose of 1.0x10^4^ colony forming units (CFU)/ mouse in 200μl sterile NaCl volume and monitored daily until they reached the experimental endpoint criteria, at which time point they were anesthetized and sacrificed. These criteria were defined as a loss of more than 20% of initial body weight, severe lethargy, absence of grooming behavior, or lack of response to external stimuli, in accordance with institutional animal welfare guidelines. The infection dose was verified by plating serial dilutions of the inoculum on BHI agar plates and counting bacterial colonies after 24 h of incubation at 37°C (applied dose approximately 10^4^ CFU).

For bacterial burden analysis, a separate cohort of mice received 5.0 × 10^3^ CFU *L. monocytogenes* intravenously. At 3.5 days post-infection, mice were anesthetized and euthanized via overdose of anesthetic. Livers and spleens were immediately harvested without prior perfusion, weighed, and homogenized in PBS using a TissueLyser II (Qiagen, Hilden, Germany). Serial dilutions of the homogenates were plated on BHI agar and incubated for 24 hours at 37°C. Bacterial burden was calculated and normalized to tissue weight (CFU/g).

For flow cytometry of intrahepatic leukocytes, mice were euthanized as described above at 3.5 days post-infection. Livers and spleens were excised, weighed and digested in RPMI medium containing 17mg/ml Liberase and 20mg/ml DNase I for 30 minutes at 37°C. Tissue digests were passed through 100μm cell strainers to obtain single-cell suspensions. Cells were then stained with fluorochrome-conjugated antibodies for 20 minutes at 4°C and analyzed by flow cytometry (Table S1 for antibody details).

Blood samples were collected from the abdominal aorta under deep anesthesia prior to euthanasia. Mice were in a fed state at the time of blood withdrawal. Collected blood was processed immediately for serum isolation by centrifugation and used for downstream analyses, including cytokine profiling and biochemical parameter measurement. Livers and spleens were harvested and either snap-frozen in liquid nitrogen for RNA/protein analyses, fixed in 4% Roti® Histofix for histology.

#### Lipopolysaccharide (LPS) injections

Age-matched (12-16 weeks) male WT and *Tgr5*^*-/-*^ mice were injected intraperitoneally (i.p.) with 22.5μg/g body weight (BW) lipopolysaccharide (LPS) derived from *Salmonella enterica* (serotype Typhimurium; Sigma-Aldrich/Merck, Darmstadt, Germany). Following injection, mice were monitored at regular intervals for signs of systemic inflammation and overall health status. Monitoring continued daily until animals reached predefined experimental endpoint criteria, which included a loss of more than 20% of initial body weight, severe lethargy, absence of grooming behavior, or lack of response to external stimuli, in accordance with institutional animal welfare guidelines.

At the experimental endpoint, mice were deeply anesthetized and euthanized by overdose of anesthetic. Blood samples were collected from the abdominal aorta while animals were in a fed state. The collected blood was immediately processed for serum isolation by centrifugation and used for downstream analyses, including cytokine profiling and biochemical parameter measurement. Livers and spleens were harvested and either snap-frozen in liquid nitrogen for RNA/protein analyses, fixed in 4% Roti® Histofix for histology.

#### Primary BMDM culture and *in vitro* stimulation

Bone marrow cells were prepared from male and female mice as previously described.^26^ Bone marrow-derived macrophages (BMDMs) were prepared by cultivating bone marrow cells for 9 days in DMEM (1000mg/l glucose) medium supplemented with 10% heat-inactivated fetal calf serum, antibiotics (100U/ml penicillin/streptomycin) and M-CSF (10ng/ml, Peprotec, Thermo Fisher Scientific). At days 4, 6 and 7 30% of the initial volume was added as fresh medium containing M-CSF (10ng/ml). After 8 days of culture, the cells were detached using trypsin/EDTA solution (incubated for 20min at 37°C). For stimulation, BMDMs were seeded in 60mm ∅ dishes or 6-well plates at a density of 5 x 10^5^ cells/dish or well and maintained overnight. Before experiments, the medium was changed to M-CSF-, FCS- and antibiotics-free culture medium. BMDMs were infected with 1 x 10^6^ CFU *L.m.* for 2 h and 6 h. For RNA sequencing analysis BMDMs were stimulated for 6 h. Extra- and intracellular ATP concentrations were determined fluorimetrically in BMDMs from WT and *Tgr5*^*-/-*^ mice infected with *L.m.* for 24 h. Supernatants and cell lysates were collected and analyzed using the ATP Colorimetric/Fluorometric Assay Kit (Sigma-Aldrich, Taufkirchen, Germany) according to the manufacturer's instructions. For inhibition experiments BMDMs were incubated with 5mM 2-Hydroxycitrate (2-HC) 30 min before additional stimulation with *L.m.* (1 x 10^6^ CFU) for 2 h and 6 h. Although dedicated toxicity assays were not conducted in this study, cells showed no reduction in viability or detectable morphological alterations at the applied concentration. For supplementation of exogenous acetate experiments, BMDMs were incubated with *L.m.* (1 x 10^6^ CFU) or LPS (100ng/ml) for 2 h followed by treatment with 10mM acetate for an additional 2 h. For glucose uptake experiments BMDMs were stimulated with Tgr5-Agonist (25μM, RO5527239 ((R,E)-1-(4-(3-(hydroxyimino)-3-(2-methylpyridin-4-yl)-1-o-tolylpropyl)phenyl)piperidine-4-carboxylic acid), Taurolithocholic acid (TLC, 25μM) or Forskolin (10μM) for 6 h, or pre-stimulated with Tgr5-Agonist (25μM) for 30 min followed by stimulation with *L.m.* (1 x 10^6^ CFU) for an additional 6 h.

For flow cytometry staining, BMDMs were incubated with Fc-block (CD16/32, BioLegend) in PBS (1:500) for 10 min on ice, followed by staining with c-Myc for 30 min on ice in the dark. Cells were washed in FACS buffer (PBS, 2% FCS, 0.5mM EDTA) and, when required, incubated with goat anti-rabbit secondary antibody (Invitrogen; 1:100) for 30 min on ice in the dark. Intracellular transcription factor staining was performed using the eBioscience™ FOXP3/Transcription Factor Staining Buffer Set (Invitrogen) according to the manufacturer’s instructions. Briefly, cells were fixed for 1 h at RT, stained overnight at 4°C in the dark with antibodies against mTOR (Invitrogen), pAMPK (Bioss), PPARγ (Bioss), Akt (Cell Signaling Technology), and c-Myc (Cell Signaling Technology). Where unconjugated primary antibodies were used, a goat anti-rabbit secondary antibody (Invitrogen; 1:1000 or 1:100 for c-Myc) was applied for 30 min on ice in the dark.

### Method details

#### siRNA transfection of BMDM cells

siGENOME SMARTpool murine Klf5 siRNA (D-062477-01; D-062477-02; D-062477-03 and D-062477-04) as well as non-targeting/scrambled siRNA (D-001210-01-05; D-001210-03-05 and D-001210-05-05, pooled) were purchased form Dharmacon (GE Healthcare, Lafayette, CO, USA). Fluorescently labeled siGlo Red Transfection Indicator siRNA was purchased from Dharmacon. Cell transfection was carried out with 4D-Nucleofector™ X Unit from Lonza (Cologne, Germany) using the software V02.16. BMDM were transiently transfected using mouse Tgr5-specific or non-targeting control siRNAs both in cotransfection with a fluorescently labeled negative control siRNA. Transfection was performed according to the manufacturer’s instructions with the P2 Primary Cell 4D-Nucleofector™ X Kit (V4XP-2024)-based protocol (D0-100). Cells were detached after 6 days of culture using trypsin/EDTA solution (incubation for 20 min at 37°C). An aliquot of cells was counted using a Neubauer counting chamber. Volumes containing 3 x 10^6^ cells were centrifuged at 200xg for 10min at room temperature and the complete supernatant was removed. The cell pellets were resuspended in 100μl room temperature 4D-Nucleofector™ solution and the siRNA mixes were added immediately (KLF5 siRNA or scrambled siRNA were mixed with siGlo Red Transfection Indicator (1μM each per reaction). After nucleofection cells were resuspended with pre-warmed medium, incubated for 10-20 min at 37°C and then prepared for FACS sorting. Cells were washed twice with cold FACS buffer (PBS-/-, 0.5 mM EDTA, 2% FCS), centrifuged (250xg for 5 min at 4°C), resuspended in 3ml FACS buffer and subsequently filtered through a 70μm Nylon Cell Strainer from Falcon (Corning, USA) into a new FACS tube to obtain single-cell suspensions. Cells were sorted using a 85μm nozzle on a FACSAria III cytometer (BD Biosciences, Heidelberg, Germany) equipped with FACSDiva 8.0 software. Using a PE filter, Cy3-labelled cells were sorted into a sterile reaction tube. Transfection efficacy as determined by Cy3-positive cells out of all counted cells ranged from 80 to 90%. Sorted cells were seeded on 60mm ∅ dishes at a density of 5x10^5^ cells/dish and culture was continued for another 24 hours.

#### Chromatin immunoprecipitation

Chromatin immunoprecipitation was carried out using the Millipore ChIP Assay Kit (Merck Millipore, Darmstadt, Germany) following the manufacturers' instructions. Briefly, the murine macrophage cell line RAW 264.7 was seeded at 1x10^6^ cells in 10cm dishes and was cultured for 1d before experiments. BMDM were generated as described above, seeded into 60mm dishes at 1 x 10^6^ cells and incubated overnight. Cells were left untreated or stimulated with *Lysteria monocytogenes* for 2 h in M-CSF-, FCS- and antibiotics-free culture medium, washed twice with PBS and replenished with medium. Nucleoprotein complexes were crosslinked with 1% (v/v) methanol-free formaldehyde (Thermo Fisher Scientific, Schwerte, Germany). Cells were washed twice with ice-cold PBS containing Protease inhibitor cocktail (Complete -EDTA, Roche, Basel, Switzerland) and collected by cell scraping. Collected cells were lysed on ice for 10 min, and the crosslinked genomic DNA was sheared by sonication on ice using a UP50H sonicator (Hielscher, Teltow, Germany) equipped with a micro tip MS1 sonotrode (3 x 40 sec pulse at 40% amplitude (cycle 1) followed by 40 sec on ice. Nucleoprotein complexes were then incubated overnight using 4μg of the following antibodies per reaction: mouse anti-Klf5, (BTEB2 (G-7); normal mouse IgG (both Santa Cruz Biotechnology, Heidelberg, Germany). Washing of protein A agarose-bound immune complexes was carried out in disposable spin columns using 600μL of each washing buffer (Thermo Fisher Scientific,). Obtained nucleoprotein complexes were decrosslinked, and DNA was purified using the QIAquick PCR Purification Kit (Qiagen, Hilden, Germany). From the purified Raw264.7 ChIP eluates, the Tgr5 promotor sequence was amplified using the Phusion High Fidelity DNA polymerase (Thermo Fisher Scientific; reaction buffer for GC-rich templates) by nested PCR using primer pair 524bp-for (5'-CCCACCTCCATCTTACATC-3') and 524bp-rev (5'-TCACATCAGCCACTCTTG-3'), followed by primer pair 334bp-for (5'-CAAGAGGGTTCTCAGAGGTAAG-3') and 334bp-rev (5'-TCTGCGGATCAAAGAGTCAC-3'). The 334bp primer pair was used for direct PCR amplification of the Tgr5 promotor region from purified murine BMDM ChIP eluates. Products were resolved on a 2.3% (w/v) TAE agarose gel or subcloned into the TOPO TA vector for amplification and subsequent sequencing of the insert.

#### Construction of promoter plasmid and luciferase assay

##### PCR was performed using the following primers specific for the murine Tgr5 promoter region

Tgr5promfor: ACGGTACCAGCAGTCAGGATGTGCCAC; Tgr5promrev: GTGCTAGCGAGTCCAAGCTGAAGGACC. The PCR product was cloned into the pGL3 basic vector (Promega, Walldorf, Germany) between KpnI/NheI sites (underlined) resulting in the promoter luciferase reporter plasmid. Alteration of the putative Klf5 binding site was performed by site-directed mutagenesis (QuickChange, Stratagene/Agilent, Santa Clara, CA). Cloning results were confirmed by sequencing and comparison to the reference sequence (NC_000067.6). The promoter constructs were transiently co-transfected with a plasmid for internal control (pRL-TK, Promega) and increasing concentrations of Klf5pMT3 or pMT3 vector control into HEK-293 cells using the transfection reagent Polyethyleneimine (PEI) (Sigma). After 24 h of transfection, cells were lysed and luciferase assays were performed using the dual-luciferase kit (Promega) according to the manufacturer's instructions.

#### Immunohistochemistry and immunofluorescence staining

Paraffin-embedded liver sections (5μm) were dewaxed, rehydrated, and subjected to antigen retrieval using citrate buffer (10mM citric acid, pH 6.0). After blocking with 2% FCS and 3% normal goat or donkey serum in PBS for 1 h at RT, sections were incubated overnight with primary antibodies against IBA1 (1:500) and CLEC4F/CLECSF13 (1:400) (see additional key resources table or Table S1). Frozen liver sections (5μm) or BMDMs (5 x 10^5^ cells/well on coverslips) were fixed with ice-cold methanol for 3 min, blocked in PBS with 3% FCS for 1h, and incubated overnight with primary antibodies against Tgr5 (1:100) and Cd11b (1:50) in a blocking solution. Fluorescein and Cyanine-3 conjugated secondary antibodies were used at dilutions of 1:100 and 1:500, respectively. Images were captured with a Zeiss LSM 880 confocal microscope (63x objective; scanning resolution: 1024x1024 pixels) or a Zeiss Axio Observer Z1/7 (40x objective; scanning resolution: 3216x2208 pixels).

#### RNAsequencing analysis

RNA QC and Library Preparation: RNA samples were measured by Nanodrop spectrophotometry and analyzed on the Agilent Bioanalyzer RNA Nano chip, which indicated RNA integrity number (RIN) >8.9 for all samples analyzed. Samples (500ng total RNA) were processed for sequencing using the GenXpro MACE Kit. The size distribution of the resulting 2 library pools was analyzed on Agilent Bioanalyzer HS DNA chips. A single library pool containing all 18 samples was generated for sequencing and quantified using the NEB Library Quant kit, a SYBRgreen-based qPCR method. The sequencing of the library pool was performed on two lanes of a HiSeq 2500 RapidRun flow cell (190122_SN552_0334_AH5YJWBCX2) in the single-read (SR) mode: 62 cycles for read 1, 9 cycles for the sample index read. Bcl-to-Fastq conversion and de-multiplexing of read1 cycle 9-62 were performed with the Illumina CASAVA 1.8.2 software using standard settings; additionally, the unique molecular identifiers (UMI) were extracted from cycles 1 to 8 of read1.

Trimming and alignment: In step 1 of the analysis, the read1 Fastq files of each sample were subjected to adapter and polyA trimming using the Trimmomatic-0.30 software tool^23^ with appropriate adapter sequences and settings. In step 2, the trimmed sequences were aligned to the Mouse Genome mm10 using the RNA aligner STAR.^27^ UMI duplicate removal: Removal of PCR duplicates from each alignment based on sequence coordinates and UMI sequence was performed by Dr. Rene Scholtysik (IFZ) using an in-house pipeline. Read filtering procedures:

In the next step, the sorted bam alignments were imported into StrandNGS (formerly AvadisNGS) and filtered on quality metrics (qm). QC-filtered bam files and the respective index were used for the following quantification. Quantification and analysis: For quantification of RNAs, qm-filtered alignments from the previous step were imported into PartekGS, which reports RPKM normalized RNA abundancies on the gene level. As an additional quality control step, a principal component analysis was performed. Subsequently, differentially expressed genes were identified using the ANOVA test implemented in PartekGS, using appropriate class labels. The step-up method for multiple testing corrections was applied to generate corrected p-values. Next, genes showing no raw count on all 18 samples were removed to yield the file. To derive lists with significantly regulated genes for each test, all genes were removed first, which did not show at least 5 raw counts in all three samples belonging to one of the two classes compared. Subsequently, genes with uncorrected p<0.05 or corrected p<0.05 were isolated. The analysis of RNA sequencing data performed nearly as previously described (4). Prior to the statistical analysis steps, the expression data were log2 transformed and normalized using quartile normalization with the 50th percentile as the reference (using edgeR). Functional analysis was conducted using the R package “clusterProfiler” and also by utilizing the Ingenuity Pathway Analysis (IPA) software (http://www.ingenuity.com/). KEGG pathway analysis, Gene Ontology (GO) and BioCarta was performed using the Database for Annotation, Visualization and Integrated Discovery (DAVID) software (https://david.ncifcrf.gov/). Gene Set Enrichment Analysis (GSEA) was performed using the Broad Institute tool GSEA v.4.3.3.

#### Quantitative real-time PCR

Total RNA was isolated from liver tissues or BMDMs using the Maxwell® 16 LEV simplyRNA Purification Kits and the Maxwell® 16 Instrument (Promega, Mannheim, Germany). 1μg RNA was reverse transcribed with the QuantiTect Reverse Transcription Kit (Qiagen) or the cDNA-Synthesis Kit (Biozym). Quantitative realtime PCR was carried out over 40 cycles on a LightCycler 480II (Roche Diagnostics, Mannheim, Germany) using TaqMan gene expression assays (Applied Biosystems, Darmstadt, Germany). The list of TaqMan assays used is provided in Table S2. Data were produced in duplicates for each gene and analyzed using the delta-delta CT method, where hypoxanthine phosphoribosyltransferase-1 (Hprt1) or Succinate Dehydrogenase Complex Flavoprotein Subunit A (Sdha) served as endogenous controls.

#### Measurement of serum parameters and cytokine levels

Blood samples were incubated for 30 min at RT to allow for agglutination, then centrifuged at 10,000 rpm for 10 min. The serum-containing supernatant was collected and stored at -80°C. Aspartate aminotransferase (AST) and alanine aminotransferase (ALT) were measured spectrophotometrically using an automated analyzer (SPOTCHEM™ EZ model SP-4430, Arkray, Inc., Kyoto, Japan).

The concentration of cytokines and chemokines in serum collected from animals 3.5 days after infection was determined using Luminex cytometric bead assay. The MCYTOMAG-70K/MILLIPLEX MAP mouse cytokine/chemokine magnetic bead panel kit was purchased from Millipore (Billerica, MA) and used according to the manufacturer’s recommendations. Samples or standards were mixed with antibody-linked magnetic beads in a 96-well plate and incubated overnight at 4°C with shaking. Plates were washed twice with wash buffer in a BioTek ELx405 washer (BioTek, Bad Friedrichshall, Germany). After 1 h incubation at RT with biotinylated detection antibodies, streptavidin-PE was added for 30 min with shaking. All incubation steps were performed at RT and in the dark. Plates were washed as above, and sheath solution was added to wells for reading in the Luminex-200 instrument. Each sample was measured in duplicates. Serum from at least 3 different animals per condition and genotype was analyzed.

#### Seahorse analyses of BMDMs

For metabolic seahorse assays, WT and *Tgr5*^*-/-*^ BMDM were stimulated and differentiated as described above. Oxygen consumption rates (OCR) and extracellular acidification rates (ECAR) were analyzed using an XFe96 Extracellular Flux Analyzer (Seahorse Bioscience). Before analysis, 2 × 10ˆ^5^ cells/well were plated in XFe96 cell culture plates coated with Cell-Tak (Thermo Fisher Scientific). As described in the manufacturer’s instructions, Seahorse XF DMEM medium was supplemented with 10mM glucose, 2mM glutamine and 1mM pyruvate (MitoStress assay medium, pH adjusted to 7.4) or 2mM glutamine and 1mM pyruvate (GlycoStress assay medium, pH adjusted to 7.4). OCR was measured under basal conditions and in response to 2.5μM oligomycin, 1.5μM fluorocarbonyl cyanide phenylhydrazone (FCCP) and 0.5μM rotenone plus antimycin A (MitoStress Test Kit, Agilent). ECAR was measured under basal conditions and in response to 10mM glucose, 2.5μM oligomycin and 50mM 2-deoxy-glucose (2-DG) (GlycoStress Test Kit, Agilent). Each sample was analyzed in technical duplicates and later averaged for independent experiments and normalized to basal ECAR using Agilent Seahorseanalytics Software (https://seahorseanalytics.agilent.com). Different OXPHOS and glycolysis parameters were calculated as described previously.^28^

#### Uptake of glucose and lipids and Glut-1 expression

Glucose uptake was measured using the fluorescent glucose analog 2-NBDG (Thermo Fisher Scientific). Primary murine bone marrow-derived macrophages (BMDM) were incubated in glucose-free RPMI medium containing 2-NBDG (100μM) for 90 min in the dark. The amount of 2-NBDG utilized by the cells was determined by flow cytometry. Uptake of short- or medium-chain fatty acids was measured by incubation with 0.5μg/mL 4,4-difluoro-5-methyl-4-bora-3a,4a-diaza-s-indacene-3-dodecanoic acid (C1 -BODIPY 500/510 C12, Invitrogen) in PBS for 20 min at RT. For long-chain fatty acid uptake, cells were incubated with 0.1μg/mL 4,4-difluoro-5,7-dimethyl-4-bora-3a,4a-diaza-s-indacene-3-hexadecanoic acid (BODIPY FL C16, Invitrogen) in PBS for 20 min at RT. For analysis of Glut-1 expression, cells were stained with Glut-1 (Thermo Fisher Scientific) in PBS for 30 min on ice after incubation with Fc-block (Thermo Fisher Scientific). After washing, cells were incubated with goat-anti-rabbit antibody (Thermo Fisher Scientific) and analyzed using flow cytometry.

#### Immunoblotting

Total protein from primary murine bone marrow-derived macrophages (BMDM) was extracted after termination of each experimental time point using lysing buffer (RL) buffer (20mM Tris pH 7.4, 140mM NaCl, 10mM NaF, 10mM NaH_2_PO_4_∗H_2_0, 1mM EDTA pH 8.0, 1mM EGTA pH 8.0, 1%Triton X-100, 1mM Na_3_VO_4_, protease and phosphatase inhibitor (Protease Inhibitor Cocktail Tablets, Roche)). Total extracted protein was quantified using Protein Assay Dye Reagent Concentrate (Bio-Rad, München) according to manufacturer’s instruction and absorbance was measured at 590 nm using a microplate reader (Tecan Safire). The lysate (protein) was mixed with sample buffer and loading dye, denatured by boiling, and then separated on 8%, 10% or 12% polyacrylamide gel. The proteins were then transferred onto nitrocellulose membranes, and blocked with 5% (w/v) nonfat dry milk prepared in Tris-buffered saline with 0,05% Tween-20 (Serva Electrophoresis) (TBST) for 1 h at room temperature. The membranes were subjected to an overnight incubation with primary antibodies (see additional key resources table or Table S1) specific to the protein of interest. Following this incubation, the membranes underwent a washing step and were subsequently exposed to secondary HRP-labeled antibodies for 1 h. Afterward, the membranes were thoroughly washed three times using TBST buffer. The membranes were then subjected to further incubation with a Western chemiluminescent HRP substrate (ECL) solution (Merk Millipore) and subsequently proteins were detected using a western blot detection machine (LI-Cor Odyssey).

To enable the re-probing of the membranes for both total protein and loading control protein, the membranes were stripped using a stripping buffer (10% SDS, 0.5M Tris pH 6.7, 2-Mercaptoethanol) for 20 min at 50°C with continuous agitation at 350 rpm. Following this, the membranes were washed for a total duration of 30 min, with each wash lasting 10 min, and subsequently re-blocked with 5% (w/v) nonfat dry milk. After the blocking step, the same procedure as described earlier was followed to detect the target protein.

For densitometry, the densitometric signal of the targeted protein was normalized to their total protein or ß-Actin and expressed as the density ratio using ImageJ tool.

### Quantification and statistical analysis

Statistical analyses and graph plotting were performed using GraphPad Prism (GraphPad, San Diego, CA, USA). Survival curves were generated using the Kaplan-Meier method and analyzed with the Logrank (Mantel-Cox) test, with results further verified by the Gehan-Breslow-Wilcoxon test. The Mann-Whitney U test, unpaired student’s t test or multiple comparisons, one-way ANOVA were used for evaluating the significance of differences between datasets as indicated in the figure legends; all data represent at least 3 independent experiments and are expressed as the mean±SEM; ∗ *p* < 0.05; ∗∗ *p* < 0.01; ∗∗∗ *p* < 0.001; ∗∗∗∗ *p* < 0.0001 were considered significant.
