# Supplementary material for: Bile acid receptor Tgr5 prevents macrophage hyperinflammation during bacterial sepsis through metabolic and epigenetic silencing
Source: iScience. 2025 Nov 5;28(12):113929. doi: 10.1016/j.isci.2025.113929 (PMC12686724; doi:10.1016/j.isci.2025.113929)
Supplement: Document S1. Figures S1–S6 and Tables S1 and S2 [file mmc1.pdf]

iScience, Volume *28*

Supplemental information

Bile acid receptor Tgr5 prevents macrophage hyperinflammation during bacterial sepsis through metabolic and epigenetic silencing

Maria Reich, Tobias Franz, Haifeng C. Xu, Paulina Philippski, Jan Stindt, Sandra Freier, Anja Sammt, Kristina Schoonjans, Philipp A. Lang, Sascha Kahlfuß, and Verena Keitel

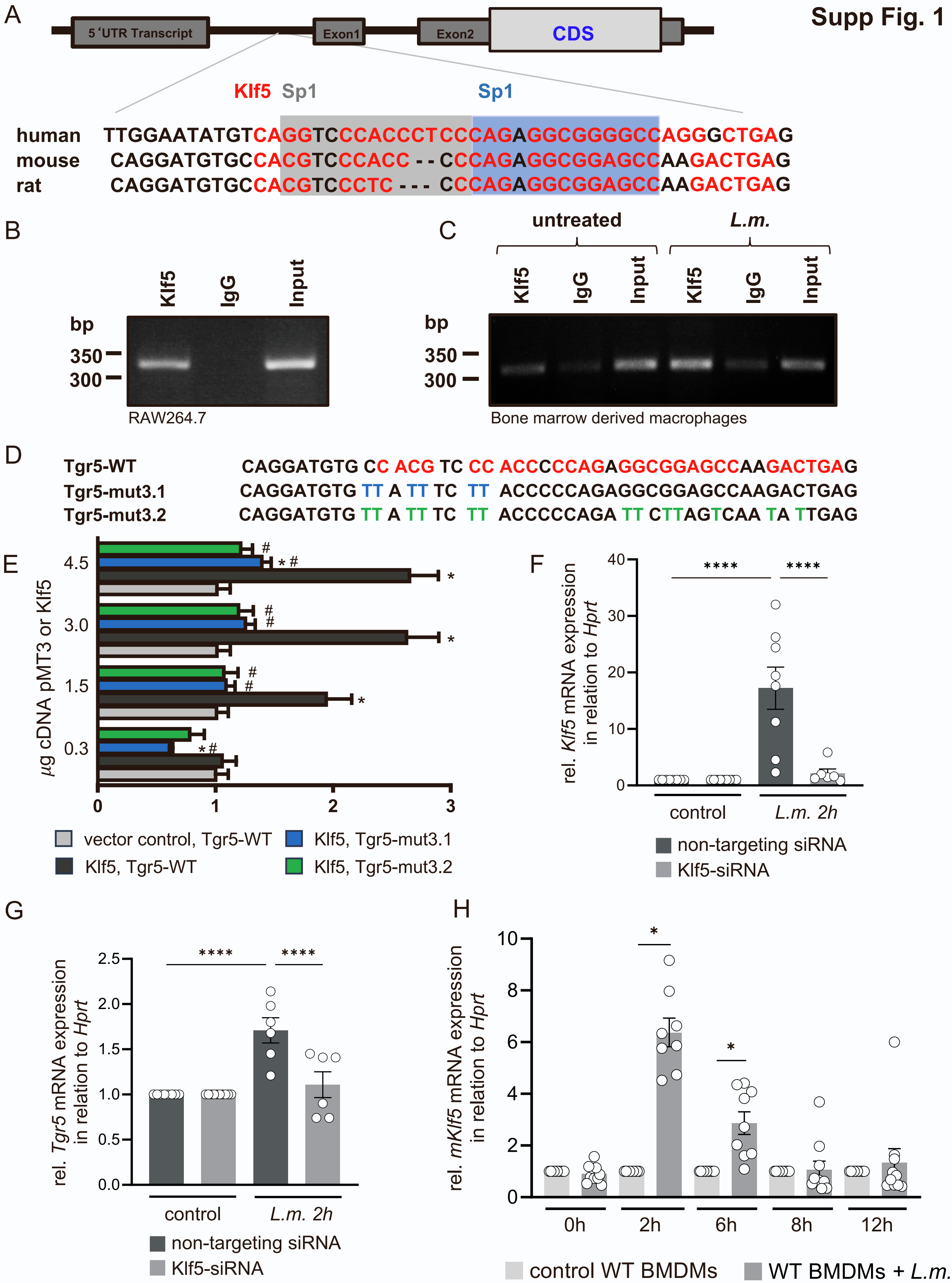
Supp Fig. 1
A
5 'UTR Transcript
Exon1
Exon2
CDS
Klf5 Sp1
Sp1
human TTGGAATATGTCAGGTCCCACCCTCCCAGAGGCGGGGCCAGGGCTGAG
mouse CAGGATGTGCCACGTCCCACC - - CCCAGAGGCGGAGCCAAGACTGAG
rat CAGGATGTGCCACGTCCCTC - - - CCCAGAGGCGGAGCCAAGACTGAG
B
Klf5
IgG
Input
bp
350
300
RAW264.7
C
untreated
L.m.
Klf5
IgG
Input
Klf5
IgG
Input
bp
350
300
Bone marrow derived macrophages
D
Tgr5-WT CAGGATGTG CC ACG TC CC ACCCCCAGAGGCGGAGCCAAGACTGAG
Tgr5-mut3.1 CAGGATGTG TT A TT TC TT ACCCCCAGAGGCGGAGCCAAGACTGAG
Tgr5-mut3.2 CAGGATGTG TT A TT TC TT ACCCCCAGA TT CTTAGTCAA TA TTGAG
E
μg cDNA pMT3 or Klf5
4.5
3.0
1.5
0.3
0
1
2
3
vector control, Tgr5-WT
Klf5, Tgr5-WT
Klf5, Tgr5-mut3.1
Klf5, Tgr5-mut3.2
F
rel. Klf5 mRNA expression in relation to Hprt
40
30
20
10
0
****
****
control
L.m. 2h
non-targeting siRNA
Klf5-siRNA
G
rel. Tgr5 mRNA expression in relation to Hprt
2.5
2.0
1.5
1.0
0.5
0.0
****
****
control
L.m. 2h
non-targeting siRNA
Klf5-siRNA
H
rel. mKlf5 mRNA expression in relation to Hprt
10
8
6
4
2
0
0h
2h
6h
8h
12h
control WT BMDMs
WT BMDMs + L.m.

Suppl. Fig. 1. In silico analysis identifies putative Klf5 binding sites in the Tgr5 promoter, and Klf5 is transcriptionally upregulated following L.m. infection or LPS exposure in wildtype mice (related to Figure 1). (A) Graphical depiction of the predicted Tgr5 promotor regions of human, mouse and rat with putative binding sites for Sp1(grey and blue boxes) and Klf5. Putative Klf5 binding sites are highlighted in red. Chromatin immunoprecipitation (ChIP) confirms of Klf5 binding to the putative Tgr5 promoter region in the RAW264.7 macrophage cell line **(B)** and in primary BMDMs **(C).** Klf5–specific antibodies were used to precipitate the transcription factor/DNA complex from sonicated chromatin. Species-matched normal IgG served as a control. **(D)** Depiction of the Tgr5 promoter sequence with targeted mutations in the putative Klf5 binding motifs. Altered nucleotides are color-coded (blue or green). **(E)** Transfection with increasing amounts of Klf5 (0.3 µg, 1.5 µg, 3 µg, 4.5 µg) results in a dose-dependent increase in luciferase reporter gene activity. Activity decreases when Klf5 binding sites are mutated. The pMT3 empty vector had no effect on promoter activity. Results are expressed as mean ± SEM, *, # indicate differences ($*p < 0.05$; unpaired student's t test) from the pMT3 empty vector control and wild-type Tgr5 promoter, respectively (vector Tgr5-WT n=16, Klf5 Tgr5-WT n=16, Klf5-Tgr5-mut3.1 n=10, Klf5 Tgr5-mut3.2 n=15). **(F, G)** Knockdown of Klf5 using siRNA significantly prevents the upregulation of Tgr5 in BMDMs following stimulation with *L.m*. Corresponding quantification of *Tgr5* mRNA levels. Data are expressed as mean ± SEM; *p* values were determined by one-way ANOVA (multiple comparisons), $****p < 0.0001$; n = 6-8 for panel F, n = 6 for panel G). **(H)** The relative *Klf5* mRNA levels in BMDMs from control and *L.m.* infected WT mice were shown in relation to *Hprt* mRNA levels (housekeeping gene). Data are expressed as mean ± SEM; *p* values were determined by Mann-Whitney U ($*p <0.05$; Mann-Whitney U test, n = 8-10).

Supp. Fig. 2

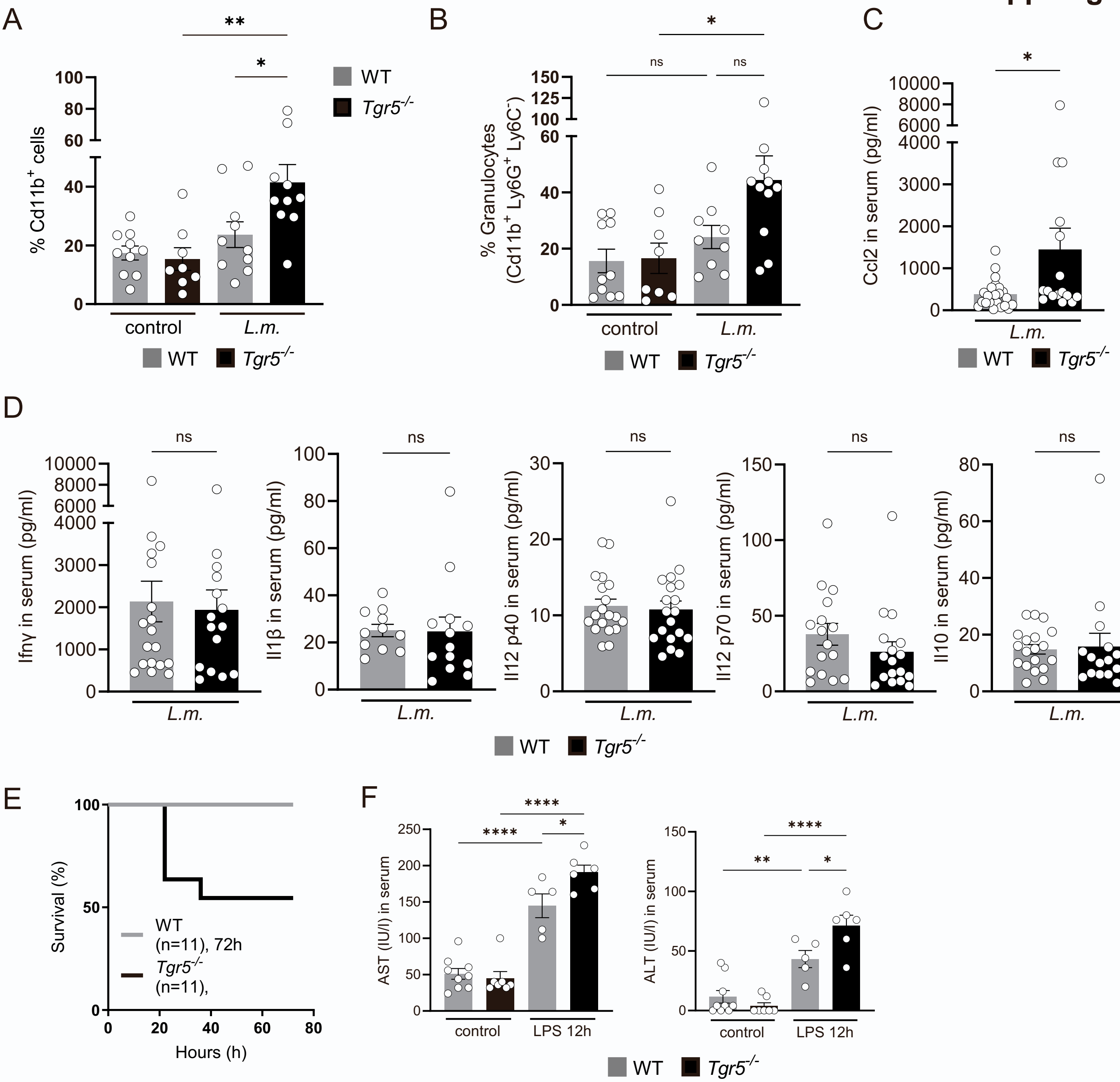

A
% Cd11b+ cells
control
L.m.
WT
Tgr5-/-
B
% Granulocytes (Cd11b+ Ly6G+ Ly6C-)
ns
C
Ccl2 in serum (pg/ml)
D
Ifnγ in serum (pg/ml)
Il1β in serum (pg/ml)
Il12 p40 in serum (pg/ml)
Il12 p70 in serum (pg/ml)
Il10 in serum (pg/ml)
E
Survival (%)
WT (n=11), 72h
Tgr5-/- (n=11),
Hours (h)
F
AST (IU/l) in serum
ALT (IU/l) in serum
LPS 12h

Suppl. Fig. 2: Absence of Tgr5 renders mice more prone to *Listeria monocytogenes* infection (related to Figure 2). Mouse liver cells were enzymatically dissociated, stained for **(A)** the common myeloid population ($CD11b^+$) and **(B)** granulocytes ($CD11b^+$ $Ly6G^+$ $Ly6C^-$) and analyzed by FACS. Data are expressed as means ± SEM, *p* values were determined by one-way ANOVA (multiple comparisons), $*p < 0.05$, $**p < 0.01$; compared to *Tgr5*$^{-/-}$ mice under control or *L.m.*-infected conditions. Sample sizes: n = 10 (WT), n = 8 (*Tgr5*$^{-/-}$) for control; n = 8-10 (WT), n = 10-11 (*Tgr5*$^{-/-}$) for *L.m.*-infected conditions. **(C/D)** Cytokine and chemokine levels were measured in the serum of WT and *Tgr5*$^{-/-}$ mice using the Luminex cytometric bead assay. Data are expressed as mean ± SEM; *p* values were determined by Mann-Whitney U test, $*p < 0.05$ compared to *Tgr5*$^{-/-}$ mice under *L.m.*-infected conditions (n = 11-23 for WT, n = 13-17 for *Tgr5*$^{-/-}$). (**E)** Kaplan-Meier survival curves showing the proportion of wildtype (WT) and *Tgr5*$^{-/-}$ mice reaching predefined experimental endpoint criteria following intraperitoneal (i.p.) injection of lipopolysaccharide (LPS; 22.5 µg/g body weight), analyzed by Logrank (Mantel–Cox) and Gehan–Breslow–Wilcoxon tests. **(F)** Serum levels of AST, and ALT. Data are expressed as mean ± SEM; *p* values were determined by one-way ANOVA (multiple comparisons), $*p < 0.05$, $**p < 0.01$, $****p < 0.0001$; compared with *Tgr5*$^{-/-}$ mice under control or LPS-treated conditions. Sample sizes: n = 9 (WT), n = 7 (*Tgr5*$^{-/-}$) for control; n = 5-6 (WT), n = 6 (*Tgr5*$^{-/-}$) for LPS-treated groups.

A

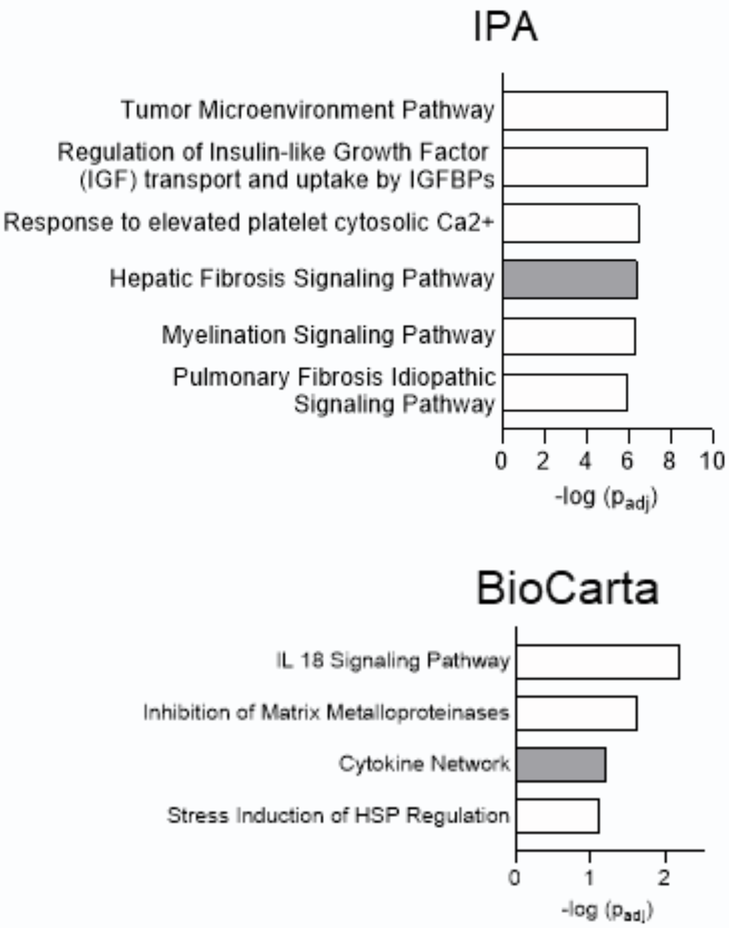

Suppl. Fig. 3: **Altered gene expression in Tgr5-deficient BMDMs links to hepatic fibrosis signaling and cytokine networks (related to Figure 3). (A)** Ingenuity Pathway Analysis (IPA) and BioCarta analysis of upstream regulated gens in Tgr5-deficient control and *L.m.*-stimulated BMDMs.

Supp. Fig. 4

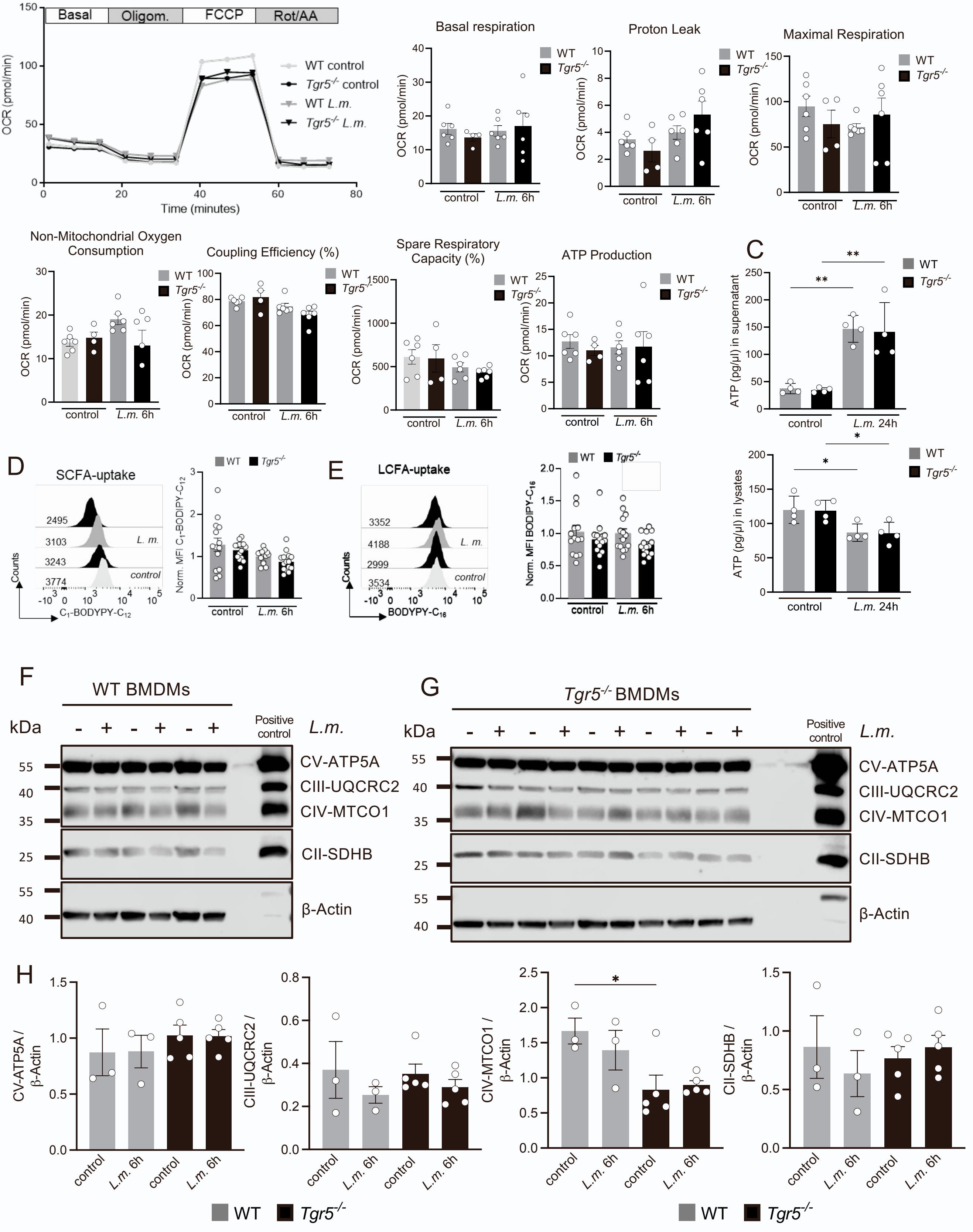

A
OXPHOS
Basal
Oligom.
FCCP
Rot/AA
OCR (pmol/min)
Time (minutes)
WT control
Tgr5-/- control
WT L.m.
Tgr5-/- L.m.
B
Basal respiration
Proton Leak
Maximal Respiration
WT
Tgr5-/-
control
L.m. 6h
Non-Mitochondrial Oxygen Consumption
Coupling Efficiency (%)
Spare Respiratory Capacity (%)
ATP Production
C
ATP (pg/µl) in supernatant
L.m. 24h
ATP (pg/µl) in lysates
D
SCFA-uptake
C1-BODIPY-C12
Counts
Norm. MFI C1-BODIPY-C12
E
LCFA-uptake
BODIPY-C16
Norm. MFI BODIPY-C16
F
WT BMDMs
kDa
L.m.
Positive control
CV-ATP5A
CIII-UQCRC2
CIV-MTCO1
CII-SDHB
β-Actin
G
Tgr5-/- BMDMs
H
CV-ATP5A / β-Actin
CIII-UQCRC2 / β-Actin
CIV-MTCO1 / β-Actin
CII-SDHB / β-Actin

Suppl. Fig. 4: TCA cycle and OXPHOS are reduced in *Tgr5$^{-/-}$* *L.m.*-stimulated BMDMs (related to Figure 4). (A) The oxidative metabolism activity of *L.m.*-infected BMDMs of WT and *Tgr5$^{-/-}$* mice was determined by Seahorse analysis. Oxygen consumption rate (OCR) was determined under *L.m.*-infected condition for 6h and uninfected conditions **(B)** Basal respiration, proton leak, maximal respiration, non-mitochondrial oxygen consumption, coupling efficiency, spare respiratory capacity and ATP production based on (A). Data are expressed as mean ± SEM, one-way ANOVA with multiple comparisons was used to determine statistical significance, defined as $*p < 0.05$ (n = 4-6). **(C)** Extra- and intracellular ATP concentrations were determined fluorimetrically in BMDMs of WT and *Tgr5$^{-/-}$* mice infected with *L.m.* for 24h. Data are expressed as mean ± SEM, one-way ANOVA with multiple comparisons was used to determine statistical significance, defined as $**p < 0.01$ (n = 4). **(D)** Uptake of short-chain fatty acid (SCFA) (BODIPY C12), **(E)** long-chain fatty acids (LCFA) (BODIPY C16) were compared between control and *L.m.*-stimulated BMDMs for 6h using flow cytometry. Data are expressed as mean ± SEM, one-way ANOVA with multiple comparisons was used to determine statistical significance, defined as $*p < 0.05$ (n = 14). **(F-H)** Western blot analysis was performed to assess the levels of Electron transfer chain (ETC) proteins in primary bone marrow-derived macrophages (BMDMs) isolated from wildtype (WT; n = 3) (F) and Tgr5 knockout (*Tgr5$^{-/-}$*; n = 5) (G) mice. Densitometric quantification was performed to evaluate the relative expression of ETC complexes (II – V), normalized to β-Actin (H). Data are presented as mean ± SEM. *p* values were determined by one-way analysis of variance (ANOVA); * $p < 0.05$.

Supp. Fig. 5

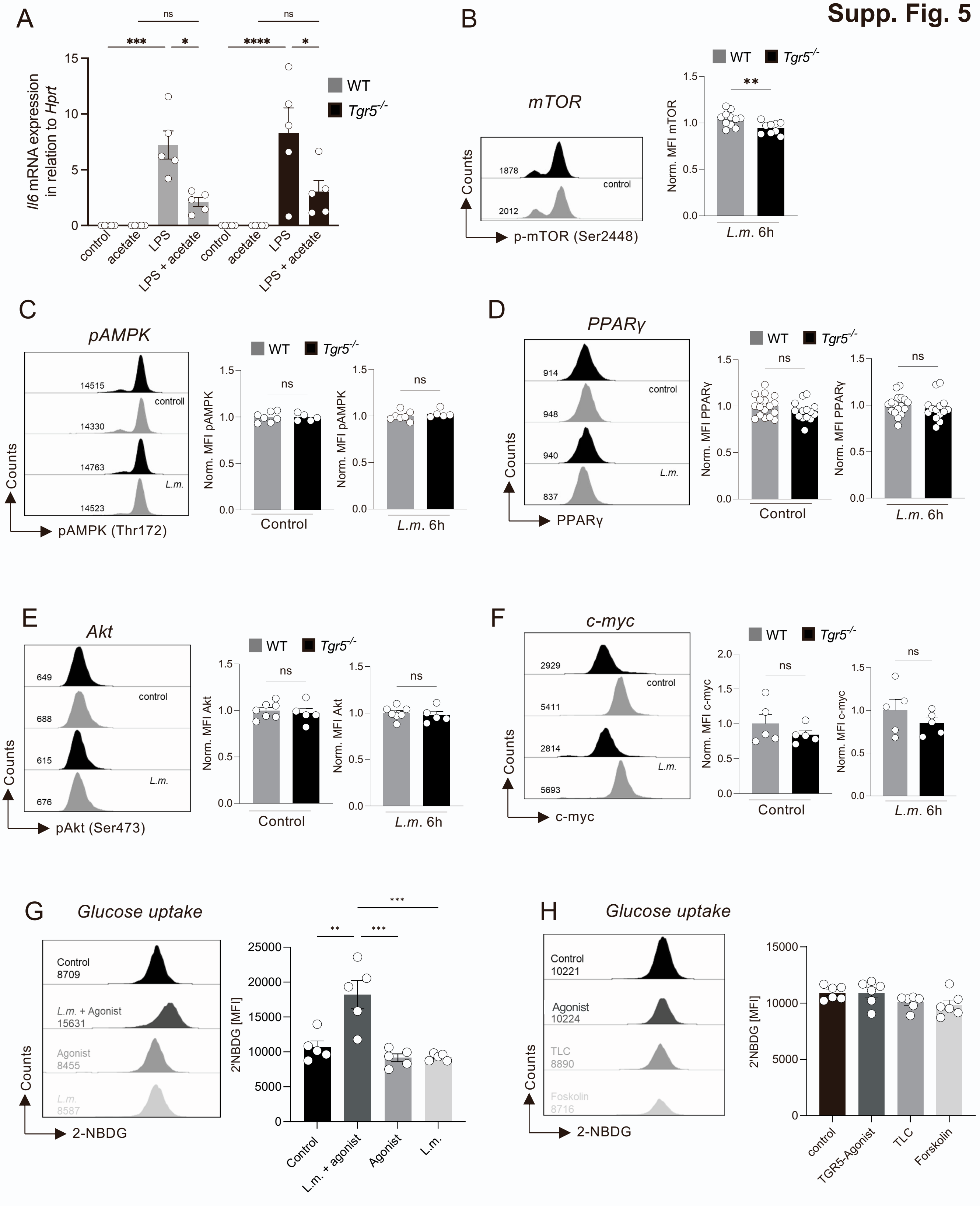

A
Il6 mRNA expression in relation to Hprt
WT
Tgr5-/-
control
acetate
LPS
LPS + acetate
ns
***
****
*
B
mTOR
Counts
p-mTOR (Ser2448)
1878
2012
control
Norm. MFI mTOR
**
L.m. 6h
C
pAMPK
14515
14330
14763
14523
controll
L.m.
pAMPK (Thr172)
Norm. MFI pAMPK
Control
L.m. 6h
D
PPARγ
914
948
940
837
Norm. MFI PPARγ
E
Akt
649
688
615
676
pAkt (Ser473)
Norm. MFI Akt
F
c-myc
2929
5411
2814
5693
Norm. MFI c-myc
G
Glucose uptake
Control 8709
L.m. + Agonist 15631
Agonist 8455
L.m. 8587
2-NBDG
2'NBDG [MFI]
Control
L.m. + agonist
Agonist
L.m.
H
Glucose uptake
Control 10221
Agonist 10224
TLC 8890
Foskolin 8716
control
TGR5-Agonist
TLC
Forskolin

Suppl. Fig. 5: Suppressive effect of acetate on infection-induced cytokine responses in WT and *Tgr5*$^{-/-}$ macrophages (related to Figure 5). (A) Effect of acetate on suppressing LPS-mediated cytokine induction in WT and *Tgr5*$^{-/-}$ BMDMs. RT-PCR analysis of *Il6* mRNA expression relative to *Hprt* in BMDMs stimulated with LPS 2h followed by treatment with 10mM acetate for an additional 2h. Data are presented as mean ± SEM. p values were determined by one-way ANOVA (multiple comparisons), * $p < 0.05$; *** $p < 0.001$; **** $p < 0.0001$, (n = 5 for WT and *Tgr5*$^{-/-}$ BMDMs). **(B)** Expression of phospho-mTOR (Ser2448) in WT and *Tgr5*$^{-/-}$ BMDMs under unstimulated conditions measured by flow cytometry. Data are shown as mean ± SEM and were normalized to the mean of WT cells; p values were determined by unpaired student's t-test, $*p < 0.05$ (n = 15-17 for WT and *Tgr5*$^{-/-}$). **(C-F)** Expression of pAMPK (Thr172) **(C)**, PPARγ **(D)**, pAkt (Ser473) **(E)** and c-myc **(F)** in WT and *Tgr5*$^{-/-}$ BMDMs under unstimulated or *L.m.*-stimulated conditions measured by flow cytometry. Data are shown as mean ± SEM and were normalized to the mean of WT cells; p values were determined by unpaired student's t-test, (n = 5-17 for WT and *Tgr5*$^{-/-}$). **(G-H)** Glucose uptake of WT and *Tgr5*$^{-/-}$ BMDMs under unstimulated (control) or *L.m.*-stimulated conditions in the presence or absence of the substances Tgr5-agonist, taurolithocholic acid (TLC) or direct stimulator of cAMP Forskolin measured by 2-NBDG using flow cytometry. Data are presented as mean ± SEM. p values were determined by one-way analysis of variance (ANOVA); ** $p < 0.01$; *** $p < 0.001$; **** $p < 0.0001$, (n = 5-6 for WT and *Tgr5*$^{-/-}$ BMDMs).

related to Supp. Fig. 4

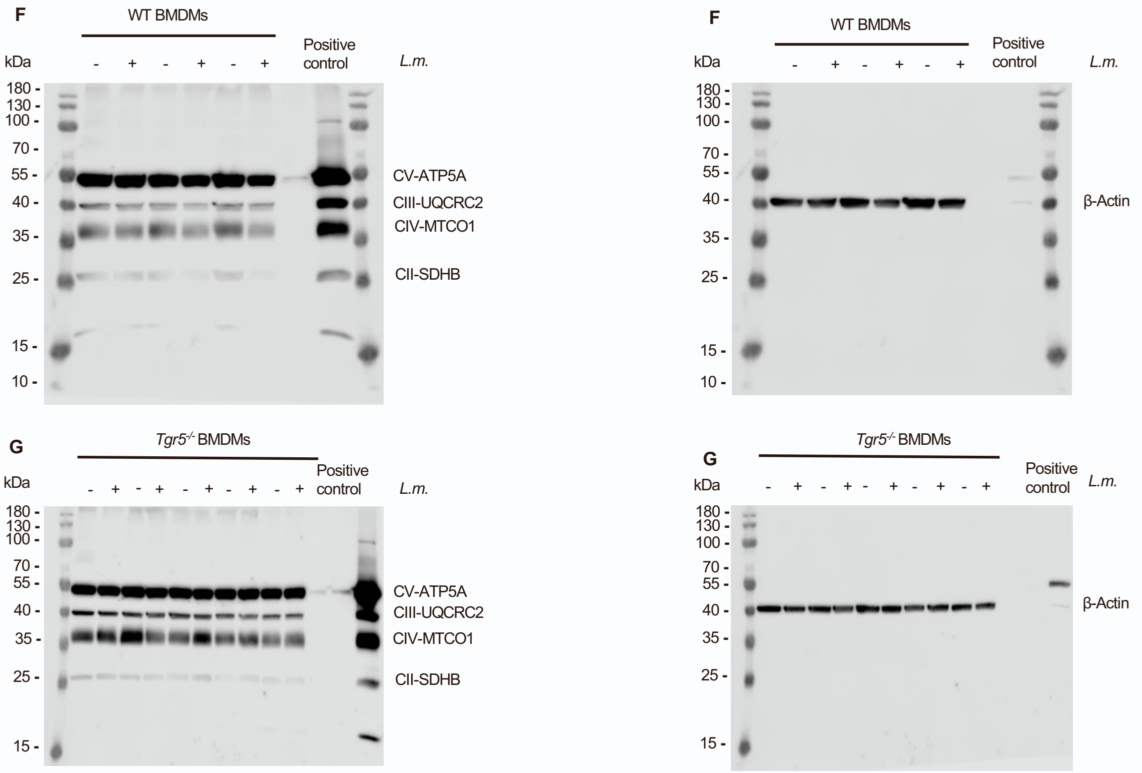

related to Figure 5

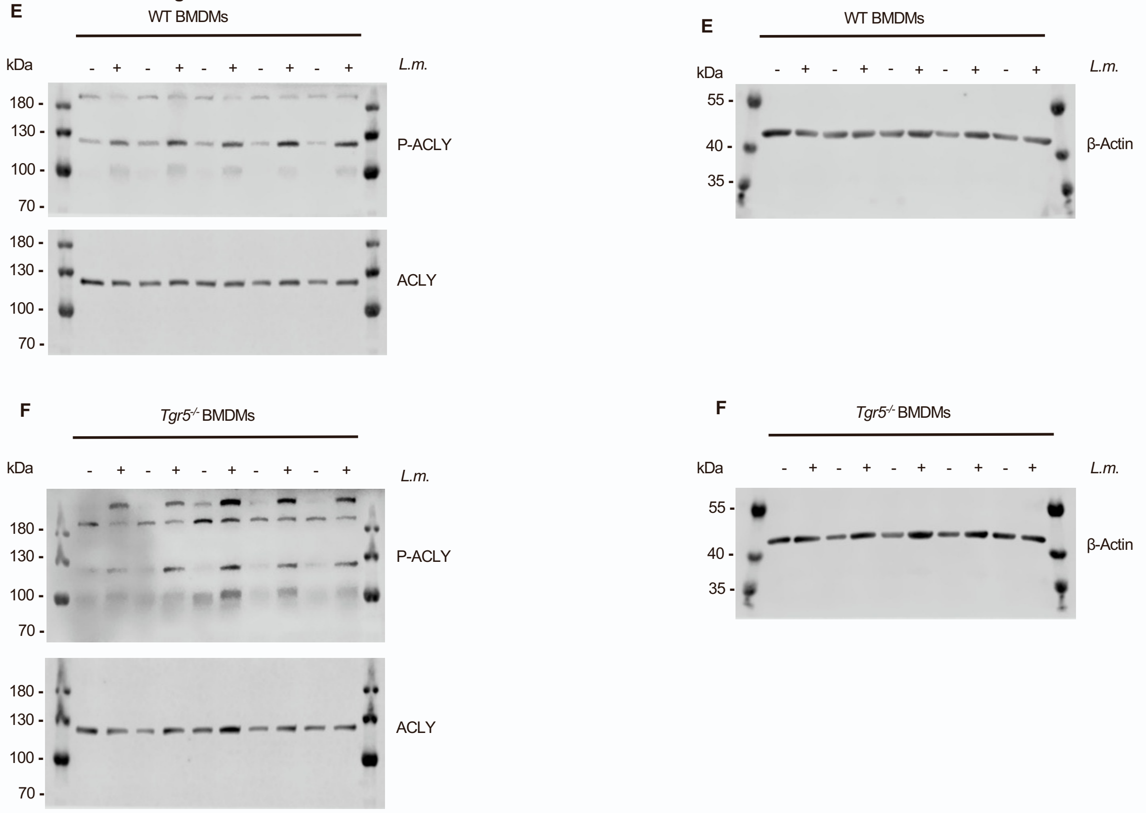

Suppl. Fig. 6: Unedited and uncropped Western blot images with the corresponding molecular weight standards

Table S1: List of antibodies List of antibodies used in this study, including target, supplier, catalog number, host species, and dilution.

Name	Supplier	Cat no.	Clone no.	Used in	Dilution
TGR5 Polyclonal Antibody	Thermo Fisher Scientific	PA5-23182		IF	1:100
Phospho-ATP-Citrae Lyase	Cell Signaling	4331		WB	1:1000
ATP-Citrate Lyase	Cell Signaling	13390		WB	1:2000
ß-Actin	Sigma	A5441		WB	1:10000
Total OXPHOS Rodent WB AB Cocktai	Abcam	Ab110413		WB	1:250
CD11b	eBioscience	14-0112-82	M1/70	IF	1:50
Phospho-mTOR (Ser2448)-PE Conjugated	Thermo Fisher Scientific	12-9718-42		Flow Cyt	1:50
Phospho-AMPK (Thr172)-FITC Conjugated	Bioss	bs-4002R-FITC		Flow Cyt	1:50
PPAR Gamma Polyclonal Antibody, PE-Cy7 Conjugated	Bioss	bs-4590R-PE-Cy7		Flow Cyt	1:50
Phospho-Akt (Ser473)-PE-Cy7 Conjugated	Cell Signaling Technology	88106	D9E	Flow Cyt	1:50
c-Myc	Cell Signaling Technology	13987	D3N8F	Flow Cyt	1:50
CLEC4F/CLECSF13	R&D Systems /Biotechne	AF2784		IHC	1:400
F4/80	Thermo Fisher Scientific	14-4801-82	BM8	IF	1:50
Glut-1	Thermo Fisher Scientific	MA5-31960	SA0377	Flow Cyt	1:500
IBA1	WAKO/VWR	WAKO-019- 19741	polyclonal	IHC	1:500
CD11b Monoclonal Antibody (M1/70), APC-eFluor™ 780, eBioscience	Thermo Fisher Scientific	47-0112-82	M1/70	Flow Cyt	1:100
Ly-6C Monoclonal Antibody (HK1.4), PerCP-Cyanine5.5, eBioscience	Thermo Fisher Scientific	45-5932-82	HK1.4	Flow Cyt	1:100
Ly-6G/Ly-6C Monoclonal Antibody (RB6-8C5), FITC, eBioscience	Thermo Fisher Scientific	11-5931-82	RB6-8C5	Flow Cyt	1:100
Cy3-AffiniPure F(ab')2 Fragment Donkey Anti-Mouse IgG (H+L)	Jackson ImmunoResearch	715-166-150		IF/IHC	1:100
Fluorescein (FITC)-AffiniPure F(ab')2 Fragment Donkey Anti-Rat IgG (H+L	Jackson ImmunoResearch	712-096-153		IF/IHC	1:100
Cy3-AffiniPure Donkey Anti-Rabbit IgG (H+L)	Jackson ImmunoResearch	711-166-152		IF/IHC	1:500
Alexa Fluor 647-AffiniPure Donkey Anti-Goat IgG (H+L)	Jackson ImmunoResearch	705-605-147		IF/IHC	1:100
Goat anti-Rabbit IgG (H+L) Highly Cross-Adsorbed Secondary Antibody, Alexa Fluor™ Plus 488	Thermo Fisher Scientific	A32731		Flow Cyt	1:100
mouse anti-Klf5, BTEB2 (G-7)	Santa Cruz Biotechnology	sc-398470-x		ChIP	
normal mouse IgG	Santa Cruz Biotechnology	sc-2025		ChIP	

Table S2: List of TaqMan gene expression assays used in this study, including target gene, assay ID, and supplier information.

**Name**	**Sequence/ Cat no.**	**Supplier**
Acly	Mm01302282_m1	Applied Biosystems
Hprt1	Mm00446968_m1	Applied Biosystems
Gpbar1 (Tgr5)	Mm00558112_s1	Applied Biosystems
IL6	Mm00446190_m1	Applied Biosystems
Klf5	Mm00456521_m1	Applied Biosystems
Sdha	Mm01352366_m1	Applied Biosystems
Tnfa	Mm00443258_m1	Applied Biosystems
